# Recent Advances of Silver Nanoparticles in Wound Healing: Evaluation of In Vivo and In Vitro Studies

**DOI:** 10.3390/ijms26209889

**Published:** 2025-10-11

**Authors:** Melis Kaya, Emir Akdaşçi, Furkan Eker, Mikhael Bechelany, Sercan Karav

**Affiliations:** 1 Department of Molecular Biology and Genetics, Çanakkale Onsekiz Mart University, Çanakkale 17100, Türkiye; mlskaya007@gmail.com (M.K.); emirakdasci@stu.comu.edu.tr (E.A.); furkan.eker@stu.comu.edu.tr (F.E.); 2 European Institute of Membranes (IEM)—UMR 5635, Univ Montpellier, ENSCM, CNRS, 34090 Montpellier, France

**Keywords:** silver nanoparticles, antibacterial activity, anti-inflammatory properties, antioxidant activity, tissue regeneration, wound healing, biomedical applications, toxicity

## Abstract

Silver nanoparticles (AgNPs) have attracted significant attention in recent years in diverse fields owing to their broad mechanisms of action. In particular, the wound healing process has become one of the main fields where the therapeutic potential of AgNPs is highlighted. AgNPs can be used as monotherapy or incorporated into composite structures in various formulations such as nanogels, hydrogels, powders, ointments, and sprays, for the treatment of a wide range of wound types including burns, excisional and incisional wounds, bone defects, surgical wounds, and diabetic ulcers. This widespread use is attributed to the strong antibacterial, anti-inflammatory, antioxidant, and cell proliferation-promoting biological properties of AgNPs. Moreover, AgNPs exhibit synergistic effects when combined with conventional antibiotics, enhancing their efficiency against resistant bacterial strains or even restoring the lost antibacterial activity. These biological properties enable AgNPs to reduce infection risk while simultaneously promoting high-quality healing by accelerating tissue regeneration. The therapeutic effectiveness of AgNPs is influenced by their physicochemical properties, including particle size, shape, and surface chemistry. In particular, synthesis methods play a significant role in determining both the biological activity and the safety profile of AgNPs. Among various methods, green synthesis approaches stand out for enabling the production of environmentally friendly, non-toxic, and highly biocompatible AgNPs. In this review, the mechanisms of action of AgNPs in wound healing are examined in detail, and recent scientific developments in this field are evaluated based on current in vitro, in vivo, and clinical studies.

## 1. Introduction

Rapid developments of nanotechnology in recent years have greatly increased the interest in functional nanomaterials in wound healing applications [1]. Nanoparticles (NPs) are among the most remarkable tools introduced by nanotechnology to science [2]. Nanoparticles exhibit special physicochemical and biological properties beyond conventional structures due to their size ranging from 1 to 100 nanometers [3].

NPs are classified into three main categories regarding their origin: organic structures, inorganic structures and carbon-based structures. NPs belonging to each category can be customized to meet different clinical needs. Inorganic NPs are generally synthesized by metals or metal oxides and are widely preferred in biomedical applications due to their chemical, structural, and multifunctional properties. This group includes metals such as gold (Au), silver (Ag), copper (Cu) as well as metal oxide NPs such as zinc oxide (ZnO), titanium dioxide (TiO_2_), and iron oxide (Fe_3_O_4_) [4,5]. Similarly, many organic and polymeric based NPs are extensively studied in such applications [6,7,8]. Among these, AgNPs are one of the most researched and used types of inorganic NPs in biomedical applications, especially in wound healing applications [9].

The AgNPs synthesis can be divided into two categories: top-down and bottom-up. Physical synthesis mediated by a top-down approach, as large metal particles are reduced to smaller sizes by physical forces. However, these methods face significant disadvantages, including high energy consumption, the requirement for expensive equipment, and challenges in controlling particle size. On the other hand, chemical synthesis methods follow a bottom-up approach, wherein molecules assemble to form larger AgNP structures. Similarly, green synthesis methods use the bottom-up approach but replace chemical reducing agents with natural reducing agents, such as microorganisms, plant extracts and biological catalysts, to reduce metal ions [10,11]. This environmentally friendly process typically yields AgNPs that are generally more stable, biocompatible, and less toxic, making them especially advantageous for biomedical applications [12].

The chosen synthesis method fundamentally defines the physicochemical profile of AgNPs, which in turn directs their biological activation mechanisms. AgNPs produced using different chemical, physical or green synthesis methods can have different shapes, sizes and surface modifications [13]. These variations influence their behavior in biological environments. For instance, smaller AgNPs, owing to their higher surface-area-to-volume ratio, tend to release silver ions (Ag+) more rapidly and efficiently, thereby enhancing their antibacterial activity [14]. As a result, broad physicochemical parameters, especially those controlling Ag^+^ release kinetics and reactive oxygen species (ROS) generation, determine the rate and degree of AgNP activation [15]. Therefore, properly understanding and managing the synthesis process ensures that AgNPs can be used both effectively and safely in wound healing.

Their substantial potential makes AgNPs a focal point of multidisciplinary research. Owing to their properties such as increased surface-area-to-volume ratios, suitability for surface functionalization, and high interaction potential in biological environments; in the current literature, many studies assess the detailed applications of AgNPs in specific fields such as food products, industry, drug delivery, wound healing, imaging, therapeutics, and so on [3]. Among these, wound healing applications of AgNPs have received significant attention in recent years.

**Figure 1:**
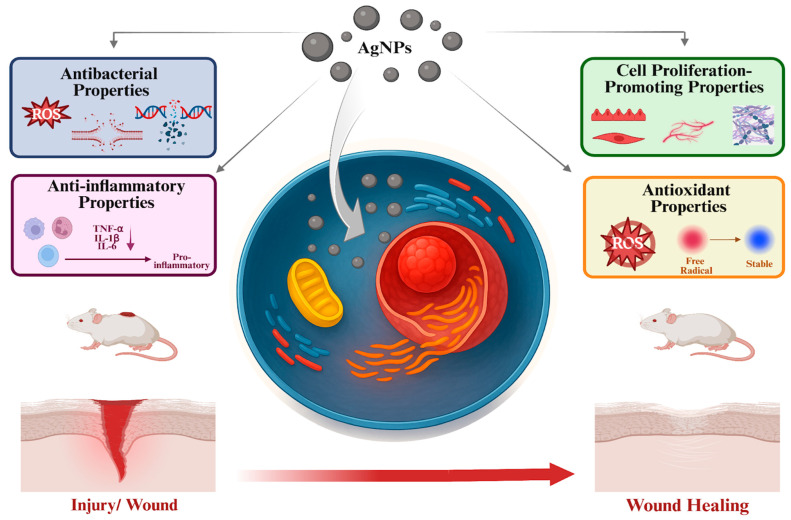
Schematic illustration of the multifunctional mechanisms of AgNPs in wound healing, including antibacterial, anti-inflammatory, antioxidant, and cell proliferation–promoting properties [18].

Wounds are pathological conditions that occur due to damage in the structural integrity of the body and require complex biological processes for healing. Silver, which has been used as a natural antiseptic against infections since ancient times, has regained attention today through its AgNP form, which further enhances its antibacterial property via modern nanotechnological [16]. However, AgNPs distinguish themselves not only for their antibacterial properties but also for their significant contribution to the wound healing process through their anti-inflammatory, antioxidant, and cell proliferation-promoting properties [17]. AgNPs contribute to wound healing through four key biological properties: antibacterial, anti-inflammatory, antioxidant, and cell proliferation–promoting properties (Figure 1). These properties are mediated by several underlying mechanisms. Antibacterial properties result from the generation of ROS, disruption of bacterial cell walls, and damage to bacterial DNA and proteins. Anti-inflammatory properties involve the downregulation of pro-inflammatory cytokines such as TNF-α, IL-1β, and IL-6. Antioxidant properties arise through the neutralization of ROS. Additionally, the cell proliferation–promoting properties of AgNPs are achieved by activating fibroblasts, keratinocytes, and endothelial cells.

In this review, the biological properties of AgNPs in wound healing are described, and their relationship with physicochemical parameters is comprehensively addressed. Examples of studies in the literature indicating that physicochemical parameters such as size, shape, and surface chemistry may alter the biological properties of AgNPs are presented. Subsequently, recent in vitro, in vivo, and clinical studies have also been reviewed to provide a broader perspective on the role of AgNPs in wound healing. The therapeutic potential of AgNPs across different wound types and various therapeutic formulations is highlighted by these studies. Beyond these therapeutic effects, current limitations such as the scarcity of long-term clinical data and the need for standardized toxicity assessments are also addressed in the review. Accordingly, this review aims to present both an evaluation of current scientific evidence and a comprehensive framework that can provide insight into future applications.

## 2. Biological Properties of AgNPs Associated with Their Wound Healing Activity

The growing scientific interest in AgNPs stems from their multifunctional biological properties, including antibacterial, anti-inflammatory, antioxidant, and cell proliferation–promoting activities. These properties support accelerated and efficient wound healing by simultaneously activating multiple mechanisms. These multifaceted biological effects make AgNPs particularly promising as therapeutic agents in treatment of difficult-to-heal wounds such as chronic wounds, diabetic ulcers, burns, surgical wounds, and other similar conditions [19].

### 2.1. Antibacterial Properties of AgNPs

**Figure 2:**
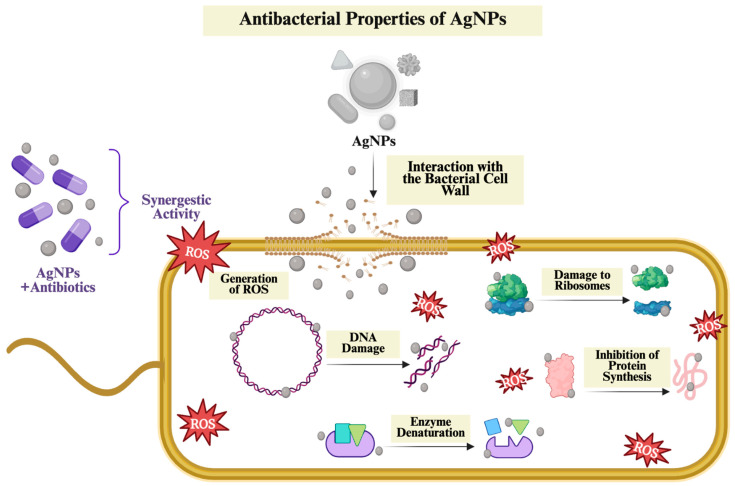
Schematic illustration of the antibacterial properties and mechanisms of AgNPs. Upon contact with bacterial cells, AgNPs disrupt membrane integrity, generate ROS, damage DNA and ribosomes, inhibit protein synthesis, and cause enzyme denaturation. Moreover, when combined with antibiotics, AgNPs can exhibit synergistic activity [21,22].

The use of antibacterial AgNPs in wound healing can provide the necessary hygienic environment for optimum wound healing by preventing any bacterial growth at the initial stage, thus contributing to the development of wound healing approaches [20]. The key antibacterial mechanisms responsible for this activity, along with the physicochemical properties influencing the antibacterial effectiveness of AgNPs, are summarized in Figure 2.

#### 2.1.1. Antibacterial Mechanism of AgNPs

AgNPs became a well-established material, primarily due to their strong antibacterial activity. Many studies have highlighted that AgNPs are significantly effective against a wide range of pathogenic bacteria. This antibacterial effect is mainly attributed to the release of Ag^+^ ions into the environment, facilitating their interaction with bacterial cells through several mechanisms [23]. According to the literature, the key mechanisms underlying the antibacterial activity of AgNPs include ROS generation, interaction with the bacterial cell wall, DNA damage and interfering with protein synthesis [24].

##### Generation of ROS

The formation of highly reactive molecules, including hydrogen peroxide (H_2_O_2_), superoxide anion (O_2_^−^) and hydroxyl radical (OH), leading to oxidative damage in cellular macromolecules, specifically protein, DNA and lipids. This oxidative damage disrupts the stability of the cell structure, interferes with biological processes, and eventually leads to cell death [25]. Smaller NPs have a larger surface area relative to their volume, allowing facilitated interaction with their surroundings and leading to high ROS production [26]. In addition, the Ag^+^ released over time from these NPs can initiate redox reactions within bacterial cells, causing further ROS formation [27]. Therefore, excessive levels of oxidative stress weaken the bacterial defense mechanisms, contributing to the bactericidal effect of AgNPs.

During wound healing, ROS act as both signaling molecules and potential sources of cellular damage. At controlled levels, ROS act as cellular messengers, organizing critical healing events such as inflammation regulation, cellular proliferation, and angiogenesis. Conversely, excessive or permanent ROS can harm surrounding tissues through oxidative stress, ultimately slowing the healing process. Thus, certain concentrations of AgNPs can facilitate wound healing by producing necessary ROS that effectively eliminate pathogens at the wound site, thus preventing bacterial interference in tissue repair [28].

##### Interaction with the Bacterial Cell Wall

AgNPs directly affect the bacterial cell wall and significantly weaken its integrity. Since AgNPs are positively charged, they can interact with negatively charged cell walls and membrane components of bacteria through strong electrostatic interactions. As a result, they can bind to the cellular membrane, causing structural and biochemical changes. This interaction negatively affects the integrity of the membrane, increases its permeability and leads to leakage of ions, proteins and organic molecules. Furthermore, this membrane damage decreases the internal pressure of the cell, leading to disruption of the osmotic balance and plasmolysis, which is the shrinkage of the cell [29].

In addition, AgNPs bind to thiol (-SH) groups in bacterial respiratory enzymes. Interaction with -SH groups leads to structural disruption and loss of function of these enzymes [30]. These changes can interfere with intracellular metabolic reactions, reduce the efficiency of energy production and cellular defense mechanisms [31]. Consequently, the combination of both structural and biochemical destruction leads to the complete elimination of the bacterium through lysis.

##### Damage to DNA and Inhibition of Protein Synthesis

This mechanism suggests that AgNPs not only penetrate and alter the structure and permeability of the cell membrane but also enter the cell to disrupt the structure and function of DNA and proteins [27]. AgNPs act as a powerful antibacterial agent by directly attacking vital intracellular macromolecules such as DNA and proteins. They bind tightly to the DNA’s phosphate backbone and to the -SH groups in proteins, disturbing their three-dimensional shape and function. Consequently, DNA replication is inhibited, leading to the suspension of cell division, while ribosomal denaturation prevents the synthesis of new proteins [24].

Moreover, Ag^+^ ions bind to nucleic acids and increase oxidative stress levels. This process causes both single and double-strand breaks, which trigger chromosomal abnormalities and genotoxic damage. On the other hand, Ag^+^ inactivates the cell’s main antioxidant defense molecule, glutathione, converting it into its oxidized form glutathione disulfide, thereby rapidly increasing the intracellular ROS levels [32]. Concurrently, increased ROS level activates the secondary mechanisms that disrupt mitochondrial membrane integrity, such as lipid peroxidation and enzyme denaturation. These secondary damages overwhelm the cell’s repair capacity, leading to cell death [33].

In conclusion, by simultaneously damaging DNA integrity and inhibiting ribosomal function, AgNPs effectively eliminate bacterial viability. While strand breaks in the genetic material prevent accurate transmission of genetic information, inhibition of protein synthesis leads to the complete interruption of cellular functions [34].

##### Effect of Antibacterial Mechanism Against Antibiotic Resistant Bacteria

Through the synergistic interplay of mechanisms such as ROS generation, disruption of bacterial cell membrane integrity, bacterial DNA damage, inhibition of protein synthesis, and inactivation of essential enzymes, AgNPs can demonstrate antibacterial activity against a wide range of microorganisms, including both Gram-negative and Gram-positive bacteria. In this way, AgNPs offer an effective therapeutic opportunity even against antibiotic-resistant strains [35].

Since antibiotic-resistant bacterial pathogens are one of the major concerns on the global health agenda, numerous research has been conducted to develop innovative antibacterial approaches [24]. AgNPs not only act alone but can also be integrated with traditional antibiotics, thereby preserving or even enhancing therapeutic efficacy even at lower antibiotic doses [36]. The multifaceted antibacterial mechanisms of AgNPs make it much more difficult for pathogens to develop resistance compared to traditional antibiotics, which typically target a single cellular function [37]. This combination can help prevent the formation of resistant bacterial populations while reducing drug-induced toxicity [27].

For instance, Khairnar et al. discussed the antibacterial efficacy of AgNPs combined with vancomycin, an extremely potent antibiotic commonly used against antibiotic-resistant pathogens [38]. The synergistic effects of the AgNP–vancomycin combination have been investigated against bacteria such as *Staphylococcus aureus* (*S. aureus*), *Enterococcus* species (VRE), and methicillin-resistant *Staphylococcus epidermidis* (MRSE), for which vancomycin or AgNPs alone exhibit limited efficacy. According to the obtained findings, the AgNP–vancomycin combination exhibited effective antibacterial activity against VRE, MRSE, and *S. aureus*, with remarkably low minimum inhibitory concentration (MIC) values of 0.1 µg/mL, ≤0.02 µg/mL, and 0.05 µg/mL, respectively. In summary, it was concluded in the discussed study that the AgNP–vancomycin combination produces a synergistic antibacterial effect, thereby improving treatment efficacy.

As another instance, Maniah et al. examined the antibacterial efficacy of AgNPs both individually and in combination with colistin or norfloxacin against bacterial pathogens [39]. Initially, AgNPs were produced with Fenugreek seed extract, and their antibacterial efficacy was examined against *Escherichia coli* (*E. coli)*, *Acinetobacter baumannii* (*A. baumannii*), *Klebsiella Pneumoniae* (*K. pneumoniae*), and *Pseudomonas aeruginosa* (*P. aeruginosa*). When AgNPs were applied at a dose of 100 µg/disk, inhibition zones were determined as 21.32 ± 0.19 mm for *E. coli*, 20.76 ± 0.31 mm for *K. pneumoniae*, 13.83 ± 0.14 mm for *A. baumannii,* and 11.26 ± 0.51 mm for *P. aeruginosa*. On the other hand, when colistin was applied alone, inhibition zones were determined as 25.18 ± 0.26 mm for *E. coli*, 20.52 ± 0.48 mm for *K. pneumoniae*, 13.89 ± 0.52 mm for *A. baumannii*, and 16.43 ± 0.18 mm for *P. aeruginosa*. When norfloxacin was applied alone, inhibition zones were recorded as 28.79 ± 0.58 mm, 24.96 ± 0.47 mm, 9.74 ± 0.26 mm, and 21.85 ± 0.34 mm, respectively. However, when the AgNP–colistin combination was applied, the inhibition zone against *A. baumannii* increased to 20.27 ± 0.58 mm, and the inhibition fold area (IFA) was calculated as 0.53. In addition, in this combination, IFA values were calculated as 0.48 for *E. coli*, 0.43 for *K. pneumoniae*, and 0.29 for *P. aeruginosa.* When the AgNP–norfloxacin combination was applied, the inhibition zone against *P. aeruginosa* increased to 27.13 ± 0.41 mm, and an IFA determined as 0.35. Furthermore, in this combination, the IFA values for *K. pneumoniae*, *A. baumannii*, and *E. coli* were determined as 0.33, 0.32, and 0.22, respectively. In conclusion, all these findings demonstrate that the AgNP–antibiotic combination achieved the highest antibacterial efficacy, even though both AgNPs and antibiotics showed strong activity individually.

Moreover, Ghaffar et al. evaluated the potential of AgNPs in restoring the effectiveness of antibiotics against drug-resistant bacteria [40]. AgNPs were synthesized with the green synthesis method using the *Nigella sativa* seed extracts and combined with four different antibiotics to evaluate their synergistic effect against MDR *S. aureus* and *K. pneumoniae* bacteria. AgNPs individually demonstrated dose-dependent antibacterial activity; at a dose of 100 µg/mL, they created inhibition zones of 23 ± 0.5 mm against *S. aureus* and 9 ± 0.7 mm against *K. pneumoniae*. The AgNP–streptomycin combination exhibited a synergistic effect by generating inhibition zones of 13 mm (130% increase) against *S. aureus*, despite streptomycin alone being ineffective, and 11 mm (110% increase) against *K. pneumoniae*. Similarly, the AgNP–cefaclor combination exhibited a synergistic effect by generating inhibition zones of 13 mm (333% increase) against *S. aureus*, despite the limited activity of cefaclor alone, and 12 mm (120% increase) against *K. pneumoniae*. The AgNP–ciprofloxacin combination showed remarkable increases in antibacterial activity by generating inhibition zones of 28 mm (133% increase) against *S. aureus* and 15 mm (150% increase) against *K. pneumoniae*, despite ciprofloxacin alone being ineffective against *K. pneumoniae*. Lastly, the AgNP–trimethoprim combination also demonstrated a synergistic effect by generating an inhibition zone of 12 mm (120% increase) on *S. aureus*, despite trimethoprim alone being ineffective, and 24 mm (240% increase) against *K. pneumoniae*. Additionally, according to the MIC analyses, the highest efficacy against *S. aureus* (0.78 µg/mL) and *K. pneumoniae* (1.56 µg/mL) was provided by the AgNP–Stp and AgNP–Tmp combinations, respectively. Ultimately, all these findings showed that, through their combination with AgNPs, antibiotics could restore their antibacterial efficacy against pathogens for which they had lost effectiveness.

To summarize, as resistance to traditional antibiotics continues to increase, AgNPs are recognized as a promising alternative, as their multiple simultaneous mechanisms of action complicate bacterial adaptation. An important aspect of this potential is the synergistic interaction of AgNPs with antibiotics, which can enhance and even restore the antibacterial efficacy of antibiotics that have become ineffective against resistant strains [36]. The antibacterial activity of AgNPs can be greatly attributed to their ability to interact with bacterial cells through multiple mechanisms simultaneously. These abilities of AgNPs are influenced by their physicochemical properties, including size, shape, and surface features. Therefore, in order to understand the antibacterial properties of AgNPs, their physicochemical properties as well as their mechanisms should be carefully evaluated.

#### 2.1.2. The Effect of Physicochemical Property of AgNPs in Antibacterial Properties

The synthesis methods and stabilizing chemicals influence the size, shape and surface features of AgNPs. These physicochemical properties are of critical importance in determining their biological activities [3].

##### Size-Dependent Effects

Among the physicochemical properties, the particle size of AgNPs has an important effect on their antibacterial activity. Smaller AgNPs, with their higher surface-area-to-volume ratio, are able to release greater amounts of Ag^+^ ions into the environment. The release of a greater amount of Ag^+^ ions enables closer contact with pathogenic bacterial cells. In this way, smaller nanoparticles generally inhibit bacteria more effectively [37].

One of the exemplary studies investigating the size-dependent antibacterial effect of AgNPs was performed by Yunping Wu et al. [41]. In this study, AgNPs with three different sizes including 2, 12, and 32 nm were synthesized by controlling the pH of the reaction. The antibacterial activity of these AgNPs was evaluated against Gram-negative *E. coli* and Gram-positive *S. aureus* using MIC, minimum bactericidal concentration (MBC), and inhibition zone measurements. As a result, the smallest AgNPs (2 nm) were found to exhibit the highest antibacterial activity.

Chen et al. synthesized AgNPs using a physical method, resulting in the formation of ultra-small AgNP populations down to the Ångström scale [42]. These 6.6 nm ultra-small AgNPs (L-AgÅPs) were compared with larger commercial AgNPs in antibacterial experiments conducted against *S. aureus* and *P. aeruginosa*. As a result, significantly stronger antibacterial activity was observed in the L-AgÅP gel formulation compared to the commercial AgNP gel.

In summary, the size of AgNPs can be adjusted through various synthesis conditions, including synthesis method, pH, pressure, AgNO_3_ concentration, and other factors. Accordingly, most studies in the literature indicated that the antibacterial efficacy of AgNPs increases as their particle size decreases [43].

##### Shape-Dependent Effects

The shape of AgNPs is a critical physicochemical determinant of their antibacterial activity, similar to their size. AgNPs can be found in various shapes, including wire-like, spherical, triangular, rod-shaped, cubic and star-shaped forms. The synthesis methods used during the production of AgNPs play a primary role in determining their shape [13,44].

For example, Hong et al. evaluated the antibacterial properties of Ag nanospheres, nanocubes, and nanowires [45]. At a bacterial density of 10^4^ CFU/mL, the MIC values were determined as 37.5 µg/mL for nanocubes, 75 µg/mL for nanospheres, and 100 µg/mL for nanowires. The strongest antibacterial effect was observed in nanocubes, which suppressed *E. coli* growth for up to 12 h at a concentration of 50 µg/mL, whereas only 6 h of suppression was observed by nanowires. As a result, although all shapes exhibited antibacterial activity, the highest activity was obtained in nanocubes, followed by nanospheres, and the lowest in the nanowire form. These results highlight the influence of NP morphology on the bactericidal efficiency of AgNPs.

Similarly, Alshareef et al. tested spherical AgNPs (AgNS) and truncated octahedral AgNPs (AgNOct) against *E. coli* and *Enterococcus faecium* (*E. faecium*) at different concentrations including 50, 100, and 1000 µg/mL [46]. Complete suppression of *E. coli* growth was shown by AgNOct at all tested concentrations, and a significant antibacterial effect was achieved by the 4th hour at the 1000 µg/mL dose. In contrast, only limited antibacterial activity was exhibited by AgNS. In the case of *E. faecium*, antibacterial activity was observed only at the 1000 µg/mL concentration of AgNOct, while no activity was observed at lower concentrations. This result provided evidence that AgNOct (1.32 m^2^/g) exhibited a stronger antibacterial effect compared to AgNS (1.26 m^2^/g) due to its larger surface area.

As a result, the shape of AgNPs can influence their contact area with the bacterial cell wall, playing an important role in their antibacterial activity [37].

##### Surface Features Dependent Effects

Surface features represent another important physicochemical property that influences the antibacterial efficacy of AgNPs. These surface features, which include stabilizers, coating agents, and functional groups attached to their surfaces, are important determinants of their antibacterial activity. Changes in these surface components may alter the release rate of Ag^+^ ions, the aggregation tendency of AgNPs, and their interaction with bacterial cell membranes [13].

Abbas et al., in a study they conducted, investigated the connection between the surface charge of AgNPs and their antibacterial activity [47]. Groups of positively, neutrally, and negatively charged AgNPs were prepared and subsequently tested against *S. aureus* and *E. coli*. It was observed that the largest inhibitory zones were formed by positively charged AgNPs. In addition, it was found that these positively charged AgNPs provided effective antibacterial activity even at much lower concentrations compared to those with neutral or negative surface charges. Electrostatic attraction allows positively charged AgNPs to bind more firmly to the negatively charged bacterial cell walls. This interaction leads to enhanced bacterial membrane disruption. Taken together, these findings suggest that surface charge may be critical for antibacterial activity by promoting stronger interaction with the bacterial membrane.

Alongside surface charge, coating AgNPs with polymers such as polyvinylpyrrolidone (PVP) or specialized synthetic coating agents exhibited a considerable increase in antibacterial activity compared with uncoated AgNPs, even at low concentrations [27].

Ashmore et al. shown growth inhibition and MIC values of uncoated AgNPs, compared with those of AgNPs coated with polyvinylpyrrolidone (Ag + PVP) and a synthetic polymer (Ag + Polymer) against *E. Coli* [48]. The results of the study showed that Ag + Polymer particles, despite containing only 10% of the amount of Ag^+^, provided up to two times more potent antibacterial activity than uncoated AgNPs. In other words, it was revealed that polymer coatings can be used to develop antibacterial systems that require lower silver concentrations while maintaining their effectiveness.

Another study was conducted by Zain et al. [49]. During the synthesis of AgNPs, chitosan was added to the solution; chitosan molecules were tightly attached to the surface of the AgNPs, providing them with a positively charged, functional coating. The resulting chitosan-coated AgNPs and controls consisting of only AgNPs were tested against *S. aureus*, *P. aeruginosa,* and *Salmonella typhimurium (S. typhimurium)*. According to the findings, bacterial colony counts were lowered by as much as 95% across all strains by chitosan-coated AgNPs, while less efficiency was observed with uncoated AgNPs under the same tested concentrations. This strong antibacterial effect is due to chitosan helping the AgNPs remain homogeneously distributed in aqueous environments and its positive charge enhances electrostatic interactions with negatively charged groups on the bacterial cell wall, which causes the membrane to break down much faster.

As a result, surface features such as surface charge, stabilizing polymers, and biofunctional coatings were shown to directly impact the antibacterial activity of AgNPs by affecting their dispersion, Ag^+^ ion release and interaction with bacterial membranes. Therefore, optimizing surface features is significant to obtain AgNPs with enhanced antibacterial properties [50].

**Figure 3:**
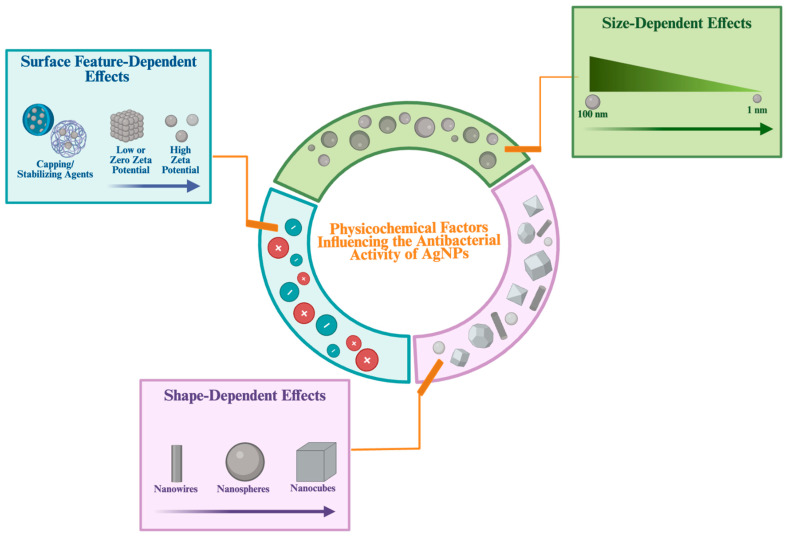
Schematic illustration of how the antibacterial activity of AgNPs is influenced by their physicochemical properties. Smaller AgNPs generally show stronger antibacterial effects due to higher surface area. The shape of the nanoparticles, such as spheres, wires, or cubes, can also affect how they interact with bacteria. Additionally, surface features like charge (zeta potential) and stabilizing agents influence how well the particles attach to and act on bacterial cells. Together, these properties determine how effectively AgNPs can damage bacterial membranes and interfere with cell function [21,22].

To summarize, physicochemical properties such as shape, size and surface characteristics influence the antibacterial activity of AgNPs by facilitating their interaction with bacterial cells and promoting cellular penetration (Figure 3). The various antibacterial activities exhibited by AgNPs, depending on the physicochemical properties and mechanisms, allow the synthesis processes to be controlled and directed toward specific targets. This also allows for the production of effective and specific targeted antibacterial agents.

### 2.2. Anti-Inflammatory Properties of AgNPs

AgNPs have attracted attention in the scientific literature not only for their antibacterial effects but also for their potential regulatory effects on the immune system. The immune system is a multifaceted defense network that protects the body against infections. AgNPs can interact with various cells of this system, such as macrophages, neutrophils and lymphocytes, resulting in diverse immunological responses. Through these interactions, AgNPs play an important role in the inflammation process, which is the first step of the wound healing process [30]. AgNPs can interact with immune cells, inducing cellular responses and modulating the immune response through alterations in the pro-inflammatory cytokine balance. These cytokines function as essential mediators that regulate inflammation and immune responses. In this respect, AgNPs are considered to actively contribute to inflammatory processes [51].

For instance, Bold et al., examined the immunological effects of AgNPs produced through the green synthesis method on a burn model in mice [52]. Following the treatment, levels of pro-inflammatory cytokines including TNF-α, IL-1β, and IL-6 were markedly reduced. In contrast, an increase in IL-10 levels was observed. These findings reveal that AgNPs may exhibit important anti-inflammatory properties that accelerate wound healing by suppressing excessive inflammation.

Similarly, Tyavambiza et al. investigated the effect of green-synthesized AgNPs on cytokine production in LPS-stimulated THP-1 macrophage cells [53]. It was observed that, in the stimulated cells, levels of IL-1β, TNF-α, and IL-6 were elevated, whereas the production of these cytokines was markedly reduced following the addition of AgNPs at a concentration of 5 µg/mL. These findings indicate the anti-inflammatory potential of AgNPs in promoting wound healing by suppressing cytokine production in stimulated macrophage cells.

Such findings in the literature suggest that AgNP administration can decrease cytokine production, thereby altering certain factors responsible for regulating immune system balance. Owing to their immunomodulatory effects, AgNPs contribute to the suppression of excessive inflammatory responses and thus promote a more regulated wound-healing process [32].

Beyond their effects in cytokine modulation, AgNPs support the wound healing process through the reduction in inflammatory damage induced by ROS [54]. ROS are generated during the inflammatory response as part of the host defense mechanism against pathogens and may cause unintended tissue damage when present at excessive levels. Research indicates that AgNPs have been shown to reduce such ROS-mediated damage by either neutralizing free radicals directly or by influencing oxidative signaling pathways in an indirect manner. This antioxidant effect serves two purposes: it protects the surrounding healthy tissues from oxidative damage and preserves the integrity of cellular structures that are vital for effective tissue regeneration.

In addition to these overall findings, evidence from several studies indicates that the anti-inflammatory effects of AgNPs may vary depending on their physicochemical properties.

As an example, Elfaky et al. compared the effects of AgNPs of different sizes (10–75 nm and 250–300 nm) on the inflammatory response in LPS-stimulated rat liver slices [55]. Within the study, it was shown that AgNPs of both size ranges significantly reduced IL-6, TNF-α, NO, and COX-2 levels, thereby exhibiting a marked anti-inflammatory effect. However, larger AgNPs were reported to be more effective in suppressing NO and TNF-α, whereas smaller AgNPs markedly decreased COX-2 expression. These results indicate that the anti-inflammatory activity of AgNPs differs according to their size.

Baganizi et al. covalently conjugated IL-10 to carboxylated PVP-coated AgNPs (Ag-PVP) to improve its bioactivity and stability [56]. In LPS-stimulated J774 macrophages, it was observed that IL-10–conjugated Ag-PVPs markedly reduced IL-6 and TNF-α levels. This conjugation was reported to exert a stronger anti-inflammatory effect compared with free IL-10, particularly at higher concentrations. Moreover, this conjugation was shown to preserve the stability of IL-10 during storage. These findings suggest that surface-functionalized AgNPs, when combined with therapeutic proteins, can enhance anti-inflammatory effects and biological stability.

In this context, Yusuf et al. modified these AgNPs through a liposomal encapsulation method to reduce the inflammatory responses they induced [57]. When tested in THP-1 macrophage cells, free AgNPs were found to markedly increase the release of pro-inflammatory cytokines, including IL-1β, IL-6, IL-8, and TNF-α. In contrast, Lipo-AgNPs were shown to significantly suppress the release of these cytokines and to modulate the inflammatory response, particularly by inhibiting STAT-3 expression. These results reveal that surface modification could be crucial in mitigating the pro-inflammatory effects of AgNPs.

Beyond these examples, the shape of AgNPs, as one of their physicochemical properties, is considered to influence their anti-inflammatory activity. However, sufficient direct comparative studies specifically relevant to this aspect are lacking in the current literature.

To summarize, thanks to all these properties and mechanisms, AgNPs play a critical role in the regulation of immune responses. Therefore, with their anti-inflammatory properties, AgNPs can be utilized as an effective agent in reducing tissue damage and accelerating the healing process, offering an effective alternative to conventional methods in wound healing applications.

### 2.3. Antioxidant Properties of AgNPs

AgNPs, which possess antibacterial and anti-inflammatory properties, also attracted attention for their antioxidant properties. AgNPs provide effective defense against bacteria through their antibacterial properties, while simultaneously reducing tissue damage through their antioxidant properties [32]. As previously explained, AgNPs demonstrate their antibacterial properties by producing ROS in target bacteria, whereas they demonstrate their antioxidant properties in the opposite manner by reducing oxidative stress in healthy tissue cells. This difference may be influenced by the nature of the target cells: AgNPs can exhibit antibacterial activity through activation of ROS production, while in mammalian cells, they might support tissue repair by potentially contributing to the neutralization of free radicals [33]. Green synthesized AgNPs might increase the protection of healthy cells from oxidative damage, potentially due to the presence of natural antioxidant molecules, such as polyphenols and flavonoids, which may contribute to free radical scavenging [58].

Lakkim et al. demonstrated the wound healing effects of green synthesized AgNPs from the leaf extracts of *Azadirachta indica* (AAgNPs) and *Catharanthus roseus* (CAgNPs) were investigated in BALB/c mice model [59]. AgNPs were found to exhibit strong radical scavenging activity in tests such as 2,2-Diphenyl-1-picrylhydrazyl (DPPH) and 2,2′-azinobis-3-ethyl benzoate-line-6-sulfonic acid (ABTS), effectively neutralizing free radicals. DPPH radical scavenging efficiency of CAgNPs was observed to be increased with concentration, showing values of 33%, 34%, 52%, 54%, and 61% at 10, 20, 30, 40, and 50 µg/mL, respectively. In comparison, AAgNPs were demonstrated to have slightly superior activities of 28%, 43%, 47%, 52%, and 70% at the same concentrations. The IC_50_ values were recorded as 36 µg/mL for CAgNPs, 35 µg/mL for AAgNPs, and 26 µg/mL for quercetin, used as the standard antioxidant control. For the ABTS tests, CAgNPs were shown to have radical scavenging efficiency of 3%, 5%, 23%, 26%, and 32% at 10, 20, 30, 40, and 50 µg/mL, while AAgNPs achieved 9%, 13%, 17%, 23%, and 40% at respective concentrations. The IC_50_ values were recorded as 45 µg/mL for CAgNPs, 43 µg/mL for AAgNPs, and 26 µg/mL for quercetin. According to these findings, the capacity of AgNPs to scavenge ROS, such as •OH, superoxide anions, and H_2_O_2_, is largely attributed to surface-associated flavonoids of plant origin. Furthermore, despite the lack of precise numerical data, a marked elevation in the activity of major antioxidant enzymes, including glutathione peroxidase (GPx), superoxide dismutase (SOD), catalase, and glutathione reductase (GR), was observed within the wound region following the administration of AgNPs. These antioxidant enzymes are essential in protecting healthy cells from oxidative damage by catalyzing the degradation of accumulated ROS in the body. Alongside these findings, although exact values were not reported malondialdehyde (MDA) levels were found to be markedly reduced in tissues treated with AgNPs. MDA is recognized as a marker of oxidative degradation of lipids that constitute cell membranes. This reduction in MDA levels indicates that the structural integrity of these membranes is preserved against oxidative damage by AgNPs. As a result, biochemical analyses at the end of day 11 demonstrated that the wound size was reduced by 93 ± 1% and 86 ± 1% in the CAgNP and AAgNP treated groups, respectively. These values were found significantly higher than those observed in the control and vehicle control groups.

Such findings demonstrate that antioxidant properties of AgNPs in wound healing can regulate oxidative damage by neutralizing free radicals, increasing the activity of antioxidant enzymes, and thereby decreasing levels of lipid peroxidation.

As another example, Qubtia et al. investigated the effects of the antioxidant and antibacterial properties of AgNPs obtained from the *Colocasia esculenta* (*Taro*) via the green synthesis method and the effects of these properties on wound healing [60]. The synthesized TCE-AgNPs were observed to have high free radical scavenging activity in both ABTS and DPPH tests, especially at concentrations ≥150 μg/mL. This observation demonstrated that these AgNPs have antioxidant properties potentially associated with chemical compounds such as flavonoids present in the plant extract from which they were synthesized. Further, the supportive effect on wound healing of TCE-AgNPs was tested using rabbit models. As a result, enhanced healing effects on rabbits treated with the TCE-AgNP group (especially the 15% TCE-AgNP film group) was observed following 14 days, which is attributed to their antioxidant and antibacterial properties. This process was found to be significantly faster and more effective compared to the standard and control groups.

In addition to plant-derived biomolecules, modifications in the physicochemical surface properties of AgNPs have also been reported to influence their antioxidant activities.

For instance, Docea et al. evaluated the effects of ethylene glycol (EG)-coated AgNPs (EG-AgNPs) and polyvinylpyrrolidone/ethylene glycol-coated AgNPs (PVP-EG-AgNPs) on the antioxidant/pro-oxidant balance in rats [61]. Following 28 days of exposure, total antioxidant capacity (TAC), thiobarbituric acid reactive substances (TBARS), and protein carbonyl (PROTC) levels, as well as GSH and CAT activities, were measured. EG-AgNPs were found to increase TAC and CAT activities and to reduce TBARS and PROTC levels. Conversely, PVP-EG-AgNPs at high doses were observed to reduce GSH and TAC levels, elevate PROTC levels, and exert a pro-oxidant effect. These findings suggest that coating agents are critical in determining whether AgNPs exhibit antioxidant or pro-oxidant properties.

Shumi et al. biosynthesized AgNPs functionalized with histidine and phenylalanine using *Lippia abyssinica* leaf extract to enhance their biological activities [62]. The antioxidant activity of the functionalized AgNPs was assessed using the DPPH assay in a concentration-dependent manner. In particular, histidine-functionalized AgNPs were determined to show the highest free radical scavenging activity. Phenylalanine-functionalized AgNPs were observed to exhibit lower activity than histidine-functionalized AgNPs, but still showed higher activity than bare AgNPs. The results revealed that surface functionalization may enhance the biological activities of AgNPs.

These studies collectively suggest that surface modifications, whether through biomolecules or synthetic coatings, are important in determining the antioxidant balance of AgNPs. However, despite the extensive evidence available on surface properties, direct comparative studies investigating the relationship between other physicochemical factors, including size and shape, and antioxidant properties are still lacking in the current literature.

Consequently, ROS generated at controlled levels during wound healing contribute to the inflammatory response as a defense mechanism to eliminate pathogens through the antibacterial properties of AgNPs. In contrast, ROS produced at excessive levels or continuously can cause oxidative damage in healthy tissues, thereby extending the healing process. At this stage, the antioxidant properties of AgNPs become crucial, and the ROS generation can be regulated with mechanisms including neutralization of free radicals, increased activity of antioxidant enzymes, and decreased levels of lipid peroxidation. Therefore, thanks to the antioxidant properties of AgNPs, oxidative damage can be reduced and tissue regeneration might be promoted.

### 2.4. Cell Proliferation-Promoting Properties of AgNPs

AgNPs attract attention due to their known antibacterial properties in the context of wound healing as well as their role in supporting cell proliferation. Cell proliferation is critical for initiating tissue regeneration and restoring structural integrity at the wound site. For this process to progress effectively, keratinocytes, fibroblasts, and endothelial cells must proliferate in a controlled manner [63]. Studies in the literature have shown that AgNPs can enhance the proliferation of cells involved in wound healing process, such as keratinocytes and fibroblasts [64].

Using a three-dimensional (3D) human skin model, Truzzi et al. examined the effects of a topical cream containing colloidal AgNPs and vitamin D_2_ [65]. The VD_2_ + AgNP cream was topically applied and significantly increased the proliferation of keratinocytes at the wound site, therefore resulting in marked closure of the wound cavity within 24 h. It was also observed that cell viability decreased to approximately 40% after the application of skin-damaging 5% sodium dodecyl sulfate (SDS). However, this reduction was only 20% when VD_2_ + AgNP cream was pre-applied. Moreover, this combination also stimulated the activity of dermal fibroblasts, supporting tissue regeneration and promoting re-epithelialization. These findings highlight the significant potential of VD_2_ and AgNPs combination which promotes cell proliferation by increasing both epidermal keratinocyte and dermal fibroblast activity.

In another example, Cheng et al. examined the effects of AgNPs that synthesized from the *Hybanthus enneaspermus* plant via the green synthesis method, on wound healing in rat models [66]. In rats that were treated with 1% HE-AgNPs ointment, hydroxyproline levels were significantly increased compared to the control group, reaching approximately 85.2 ± 2.4 μg per 100 mg of tissue. Considering the histological findings, it was observed that HE-AgNPs primarily increased fibroblast activity, which in turn stimulated cell proliferation and led to an increase in collagen synthesis. Thanks to these effects, including the contribution of antibacterial, anti-inflammatory, and antioxidant properties, the wound closure rate in rats treated with HE-AgNPs ointment was observed to exceed 95% on day 21, while this rate was reported to be approximately 70% in the control group. These findings provide evidence that HE-AgNPs promote cell proliferation by increasing fibroblast activity and thus accelerating the wound healing process.

AgNPs support tissue repair by stimulating the proliferation of essential epidermal and dermal cells, specifically keratinocytes and fibroblasts [51]. At the same time, AgNPs promote the regeneration of the extracellular matrix by stimulating collagen production at the wound site, which strengthens the structural integrity of the regenerating tissue [32]. Moreover, AgNPs promote the creation of new blood vessels by supporting the process of angiogenesis, thereby providing the regenerating tissue with the essential oxygen and nutrients [67]. Furthermore, AgNPs may prevent excessive or chronic inflammation by modulating the inflammatory response, thereby establishing a more favorable environment for efficient wound healing [9].

At the same time, the few studies available in the literature suggest that the proliferative effects of AgNPs are not a fixed property and may vary with the physicochemical parameters of the nanoparticles used. As a notable example, Orlowski et al. aimed to evaluate the importance of size in this context by comparing the effects of tannic acid (TA)-modified and unmodified AgNPs on the main biological processes related to wound healing [68]. In the in vitro keratinocyte scratch assay, which primarily measures cell migration and is closely linked to proliferative responses during re-epithelialization, TA-AgNPs were reported to significantly increase cell migration, whereas the increase with unmodified AgNPs was not statistically significant. In the in vivo mouse splint wound model, treatment with 33–46 nm TA-AgNPs was found to accelerate re-epithelialization, concomitantly with a marked increase in angiogenesis and granulation tissue formation. By contrast, with 13 nm TA-AgNPs, a delay in the proliferative phase and a slowing of wound closure were observed. Furthermore, the mRNA levels of VEGF-α, PDGF-β and TGF-β1, which promote proliferation, were detected to be elevated at an early stage in all AgNP groups. These findings suggest that the effect on proliferation and tissue regeneration depends on both the nanoparticle size and the surface properties. Although the current findings are valuable, there is still a lack of experimental studies directly comparing the effects of the physicochemical properties of AgNPs on proliferation in fibroblast and keratinocyte cells. Therefore, in future studies, comprehensive evaluation of parameters such as size, shape, and surface modification in relation to proliferation-promoting effects may contribute to a better understanding of these biological mechanisms.

To summarize, AgNPs may improve tissue regeneration by promoting cell proliferation. They also support wound healing through mechanisms involving vascularization, extracellular matrix remodeling, and immune regulation. As previously described, beyond their role in promoting cell proliferation, AgNPs also contribute to wound healing through their antibacterial, antioxidant, and anti-inflammatory properties. Each of these properties complement the others and functions concurrently on different phases of wound healing, thereby markedly accelerating and enhancing the overall wound healing process.

## 3. Wound Healing Activity of AgNPs in In Vitro and In Vivo Models

**Figure 4:**
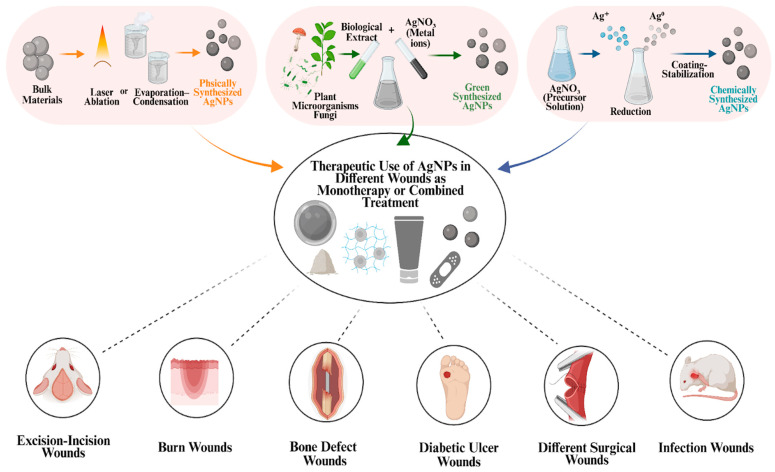
Schematic overview of AgNPs produced through different synthesis methods, their various therapeutic formulations, and their applicability across different wound types [19,70].

Recent investigations on the wound healing capacity of AgNPs have revealed the diverse therapeutic potential of these nanomaterials. Their distinctive physicochemical properties, such as high surface-area-to-volume ratio, shape, and adaptable surface chemistry, support antibacterial, anti-inflammatory, antioxidant, and cell proliferation-promoting activities. Moreover, these physicochemical properties can be adjusted through various synthesis methods, including physical, chemical, and green synthesis. In this way, the selected synthesis method influences the biological behavior of AgNPs in the wound environment. As shown in Figure 4, AgNPs synthesized by these methods can be used either as monotherapy or as part of combined treatments by being incorporated into various formulations such as hydrogels, ointments, sprays, powders, and dressings. Through these various combined treatments or when used as monotherapy, AgNPs have been evaluated as therapeutic agents in a wide range of wound models, including excision/incision wounds, burn wounds, bone defects, diabetic ulcers wounds, surgical wounds, and infected wounds (Figure 4). Due to their potential wound healing–supporting effects, AgNPs have become a significant focus of research and have been systematically investigated in both in vitro and in vivo studies. Subsequent clinical studies have also investigated the wound healing potential of AgNPs [69].

### 3.1. In Vitro Studies with Cell Culture Models

In vitro studies conducted under laboratory conditions are extremely important for understanding the effects of AgNPs on wound healing. Since these studies are conducted independently of the complex physiological environment of a living organism, they provide an opportunity to examine cellular processes in a simpler and more controllable setting. These studies allow researchers to determine how AgNPs interact with different cell types, which molecular pathways they activate, and how they modulate cellular behavior. In the context of these studies, one of the most commonly used methods is the wound healing (scratch) assay. In this method, a controlled scratch is usually created in a confluent cell monolayer by mechanical, thermal, or chemical means. Subsequently, the closure of the scratch over time is monitored to assess cell migration and proliferation [71].

Tyavambiza et al. investigated the in vitro wound healing effects of AgNPs, green synthesized from aqueous *Cotyledon orbiculata* extracts (Cotyledon-AgNPs), using a scratch assay [72]. A mechanical wound model was established by scratching the surfaces of keratinocyte (HaCaT), epithelial (CHO), and fibroblast (KMST-6) cells after 24 h of incubation. In this wound model, two treatments were applied: 2.5 µg/mL Cotyledon-AgNPs and 15 µg/mL aqueous extract. Additionally, 15 µg/mL allantoin was used as a positive control, while cells receiving no treatment were used as the negative control. The obtained data demonstrate that both Cotyledon-AgNPs and the aqueous extract significantly accelerated wound closure in all cell types compared to the negative control. In treated cells, complete wound closure was observed in HaCaT at 48 h, in CHO at 72 h, and in KMST-6 at 96 h. These findings show that each cell type has different migration rates and motility. In HaCaT cells, almost complete wound closure was observed within 24 h following treatment with Cotyledon-AgNPs. In addition, Cotyledon-AgNPs exhibited higher wound healing activity than allantoin in HaCaT and CHO cells. In conclusion, these findings indicate that Cotyledon-AgNPs, even at low concentrations, support wound healing by promoting both cell proliferation and migration.

In another in vitro study, Vijayakumar et al. evaluated the antibacterial, antioxidant, and wound-healing properties of AgNPs biosynthesized from probiotic bacteria [73]. Initially, among the probiotic bacteria isolated from cow and buffalo milk, *Lactiplantibacillus plantarum* was identified as the strain showing the highest antibacterial activity. Owing to this property, this strain was used to synthesize AgNPs with an AgNO_3_ solution. The synthesized AgNPs were spherical with an average diameter of 14 ± 4.7 nm, and a surface plasmon resonance (SPR) peak was observed at 425 nm. The wound-healing effect of these AgNPs, which had previously been tested for antibacterial and antioxidant activities, was tested in vitro. A wound model was created by scratching Vero cell monolayers. The cells were then treated with AgNPs at a concentration of 60 µL/mL, and cell migration was observed over time. Following the treatment, wound closure was measured at 47% after 24 h, increasing to 66% at 48 h, and reached 96% at 72 h. Overall, the findings from this in vitro study provided evidence that AgNPs strongly promote fibroblast cell proliferation and migration, thereby significantly accelerating the wound healing process Consistent with previous reports, these wound closure effects are thought to be partly mediated by the antibacterial and antioxidant properties of AgNPs. By lowering microbial burden and oxidative stress, AgNPs are considered to create a favorable microenvironment for cell migration and proliferation. AgNPs have also been proposed to stimulate fibroblast-to-myofibroblast differentiation, thereby contributing to wound contraction and the initiation of the proliferative phase.

As another example, Muthukumar et al. evaluated the antibacterial properties and wound healing effects of a nano-herbal ointment composed of *Tridax procumbens* extract, gelatin stabilized AgNPs (G-AgNPs), and rhamnolipid biosurfactant obtained from *P. aeruginosa* [74]. The synthesized G-AgNPs were determined to be spherical with sizes ranging from 10 to 30 nm. Initially, the antibacterial activity of the prepared ointment was assessed against *S. aureus* and *E. coli*. At a concentration of 100 µg/mL, inhibition zones of 19.5 mm and 15.5 mm were measured, respectively. Following the assessment of its antibacterial activity, the wound-healing potential of the ointment was also evaluated in vitro. A wound model was created by scratching the surface of Vero cells with a pipette tip, and then 100 µg/mL of the ointment was applied. After 72 h of incubation, a wound closure rate of 90 ± 2% was recorded in the treated group, compared with 62 ± 2% in the control group. As a result, the findings from this in vitro study demonstrated that the ointment containing G-AgNPs, biosurfactant, and *Tridax procumbens* extract markedly accelerated wound closure and cell migration.

As a further example, Vijayakumar et al. investigated the green-synthesized AgNPs from macrofungus *Phellinus adamantinus*, demonstrating their antibacterial activity and effects on wound healing [75]. The synthesized AgNPs were determined to be spherical with an average size of 50 nm, and an SPR peak was observed at 423 nm. Initially, the antibacterial activity of the synthesized AgNPs was tested against pathogenic bacteria, including *S. aureus*, *Bacillus subtilis* (*B. subtilis*), *E. coli*, and *P. aeruginosa*. Strong antibacterial activity against *S. aureus* was demonstrated by the AgNPs, with an inhibition zone of 11 mm. The MIC for all tested bacteria was determined to be 3.125 µg/mL. After confirming the antibacterial activity, the wound healing potential of the composite nanofibers containing AgNPs, PGA, and PVA was evaluated in vitro. In a wound model created by scratching the surface of Vero cells, 20% wound closure was observed after 24 h following the application of the AgNP–PVA nanofiber. This outcome indicated greater healing efficacy compared to the negative and positive controls. Consequently, the findings from this in vitro study provided evidence that AgNP–PVA nanofibers supported wound healing by promoting cell migration and proliferation.

To summarize, as observed in the example studies presented above, various in vitro studies demonstrate the wound healing promoting effects of AgNPs at the cellular level. In addition to these examples, recent in vitro studies published after 2020 are summarized in Table 1. These in vitro findings suggest that AgNPs, through their antibacterial, anti-inflammatory, and antioxidant properties, not only provide an efficient healing environment but also stimulate cell proliferation at the wound site, accelerate re-epithelialization, and enhance cellular repair. Moreover, these findings from in vitro studies form the basis for in vivo studies and contribute to the development of future clinical applications.

### 3.2. In Vivo Wound Healing Models

In vivo studies conducted on living organisms under physiologically realistic conditions are of great importance for understanding the effects of AgNPs on wound healing. These studies allow researchers to determine how AgNPs affect the multifaceted interactions within the organism during the wound healing process in a real wound environment. In contrast to in vitro studies, which are typically limited to simplified 2D models, in vivo studies reflect the complexity of physiological conditions, including stromal cells, extracellular matrix, and systemic responses. Therefore, these models help provide a more realistic representation of the wound healing process by building on the knowledge obtained from in vitro studies. The effect of AgNPs on wound healing has been through highlighted numerous in vivo studies conducted using different animal models. The selected examples presented in this section extensively demonstrate the multifunctional roles of AgNPs in enhancing wound healing across different wound models.

#### 3.2.1. Mice and Rat Models

Mice and rat models are commonly used living organisms in many studies examining the effects of AgNPs on wound healing. In such in vivo studies, controlled wounds such as burns, excisions and incisions are created on the skin of mice or rats, treatment methods containing AgNPs are applied to these wounds and the healing process after treatment is analyzed.

Gaikwad et al. investigated the wound healing effect of a nano-gel containing AgNPs synthesized via a green method from the fungus *Fusarium oxysporum* in albino Wistar rats [86]. Initially, the obtained AgNPs were incorporated into Carbopol-based nano-gels at concentrations of 0.1, 0.5, and 1 mg/g. Then, these AgNP-containing nano-gels were applied to male albino Wistar rats, on which controlled models of incision, excision, and burn wounds were created, and the healing process of these wound types was observed. In the incision model, the greatest tensile strength on day 10 (380.33 ± 2.0 g) was reached by the 0.5 mg/g AgNP nano-gel group. In the excision model, the highest wound closure rate of 98.30% ± 1.43 on day 20 was recorded for the 0.1 mg/g AgNP nano-gel group, whereas in the burn model, the highest rate of 98.60% ± 2.41 on day 15 was observed with the 1 mg/g group. These findings suggest that the optimal concentration of AgNPs varies depending on the wound type. Moreover, superior wound healing performance was observed in all models treated with AgNP-containing nano-gels compared with both the gel-only and untreated control groups. In conclusion, the findings from this in vivo study provided evidence that AgNP nano-gel treatment enhanced wound healing in different wound types, with no observable toxic effects detected at the tested concentrations.

Francisco et al. investigated a Pluronic^®^ F127 hydrogel containing AgNPs for its wound-healing effect in burn wounds of albino CD-1 mice [87]. Initially, AgNPs were produced via a chemical synthesis method, and they were characterized with an average size of 48.04 ± 14.87 nm, a zeta potential of −0.79 ± 2.17 mV, and an SPR peak at 407 nm. These AgNPs were incorporated into a Pluronic^®^ F127 hydrogel, whose thermoresponsive property enables local, controlled, and sustained release by retaining nanoparticles at the site of application. After AgNPs were incorporated into the Pluronic^®^ F127 hydrogel, in vitro tests were performed to test their antibacterial activities against *E. coli*, *P. aeruginosa*, and *S. aureus*. Subsequently, the effect of this AgNP-containing hydrogel on the healing process of controlled, chemically created burn wounds in albino CD-1 mice was investigated through an in vivo study. Three different treatment groups were applied to the created burn wounds: the first treatment group received AgNP-hydrogel (3.3 pmol/cm^2^), the second group was treated with silver sulfadiazine cream (15.3 µmol/cm^2^) as a positive control, and the third group was designed as a negative control using Pluronic^®^ F127 alone. Although weight loss was observed in all treatment groups following burn wounds, the AgNP-hydrogel group showed improved healing compared to the other groups by regaining 96% of their body weight, demonstrating enhanced wound closure and minimal scarring. Moreover, based on the obtained data, although the second treatment group, AgSD cream, showed a similar rate of wound closure, it was observed that the AgNP-hydrogel achieved this effect at significantly lower doses. In conclusion, the findings from this in vivo study provide evidence that AgNPs incorporated into Pluronic^®^ F127 hydrogel significantly enhance wound healing in burn wound models, even at low concentrations.

In another example, Haidari et al. investigated the wound healing effect of a hydrogel composed of AgNPs incorporated into Pnipam-PAA in infected burn wounds in BALB/c mice [88]. Initially, AgNPs were produced through a chemical synthesis method, and their average size was determined to be approximately 2.95 ± 0.68 nm. These AgNPs were incorporated into the Pnipam-PAA hydrogel, which provides targeted ion release at the infected site on demand, thanks to its pH- and temperature-sensitive structure. As the AgNPs were incorporated into Pnipam-PAA, in vitro experiments were conducted It was determined that AgNP-hydrogel exhibited over 95% cell viability in human fibroblast (HFF) and (HaCaT) cells, and also showed over 95% antibacterial activity against *S. aureus*, *S. epidermidis*, and *P. aeruginosa* bacteria. Subsequently, partial-thickness scald burns were created on the dorsal region of BALB/c mice by hot water bath exposure (65 °C for 45 s), and after 48 h, a bioluminescent *S. aureus* Xen29 (1 × 10^7^ CFU) inoculation was performed on the area to establish an infected burn model. Three different treatment groups were applied to these infected burn wounds: hydrogel alone (negative control), AgSD cream (positive control), and AgNP-hydrogel. On day 12, the wound area in the AgNP-hydrogel group was reduced to 17%, while it was 22% in the positive control and 28% in the negative control. Moreover, the re-epithelialization rate was measured at 75% in the AgNP-hydrogel group, compared to 62% in the AgSD cream group and 61% in the negative control. As a result, the findings from this in vivo study provide evidence that AgNPs incorporated into the Pnipam-PAA hydrogel significantly enhance wound healing in an infected burn model, not only by ensuring infection control but also by supporting tissue repair processes in a multifaceted manner, even at low concentrations.

As another example, Maheshwari et al. investigated the wound healing effect of AgNPs obtained from the flower extract of *Woodfordia fruticosa* (Wf) and incorporated into Carbopol 934 gel on excision wounds in female Swiss mice [89]. Initially, AgNPs were produced with green synthesis using flower extract of Wf, and it was determined that these AgNPs had spherical morphology with an average size of 5 nm and a zeta potential of −0.283 mV. The antibacterial activity of Wf-AgNPs was observed against *S. aureus*, *E. coli*, and *Salmonellatyphi* using the disk diffusion method. Subsequently, three different treatment groups were applied to the created sterile excision wounds: the first group was treated 1% WfAgNPs incorporated into Carbopol 934 gel, the second group was treated with a gel containing only 1% Wf flower extract as a positive control, and the third group was designed as a negative control with no treatment. According to the analyses obtained on day 21, a significant increase in collagen deposition and granulation tissue formation, along with a decrease in the number of inflammatory cells, was observed in the group treated with WfAgNPs-Carbopol 934 gel. In contrast, pronounced inflammation was evident in the untreated control group, while partial wound healing was recorded in the group treated with the Wf flower-extract gel. Moreover, in skin irritation tests, the lowest score (1.200) was recorded for the WfAgNPs-Carbopol 934 gel group, compared with 5.200 for the negative control group and 3.400 for the positive control group. Overall, the findings from this in vivo study provide evidence that AgNPs synthesized from *Woodfordia fruticosa* and incorporated into Carbopol 934 gel enhance wound healing in a sterile excision wound model and promote higher-quality tissue regeneration.

In another example, El-Hamid et al. evaluated the wound healing effect of a hydrogel composed of AgNPs synthesized from *pomegranate peel* extract (PPE), which were incorporated into hyaluronic acid (HA) and carboxymethylcellulose (CMC), on infected excision wounds in Sprague Dawley rats [90]. Initially, the AgNPs synthesized from pomegranate peel extract by green synthesis were determined to be spherical in shape. In vitro experiments were conducted with these PPE-AgNPs, and they were found to have exhibited significant antifungal activity against *Candida albicans* (*C. albicans*) strain, as demonstrated by a 34 mm inhibition zone in the agar diffusion test and MIC/MFC values of 1.25 and 2.5 µg/mL, respectively. Subsequently, these PPE-AgNPs were incorporated into a hydrogel containing CMC and HA, which provides controlled release properties thanks to its high water retention and swelling capacity. After the nanocomposite PPE-HA-AgNP hydrogel was obtained, 2 mm excision wounds were created on the dorsal region of male Sprague-Dawley rats, and three days later, the wounds were inoculated with *C. albicans* (10^7^ CFU/mL) to establish an infected excision wound model. Different treatment groups were applied to the infected excision wounds: a negative control group with no treatment, a positive control group treated with 2% ketoconazole cream, and treatment groups receiving PPE-HA-AgNP hydrogel at three different concentrations (25%, 50%, and 100%). On day 21, the wound area in the group treated with 100% PPE-HA-AgNP hydrogel was reduced by over 85%, whereas this reduction was observed to be 79% in the positive control group and 80% in the group treated with 50% PPE-HA-AgNP hydrogel. Moreover, a significant reduction in fungal load, measured as 2.95 log_10_ CFU/g, was observed by day 15 in the group treated with the hydrogel containing 100% PPE-HA-AgNPs. Even though the 100% PPE-HA-AgNP hydrogel demonstrated the strongest effect, the 25% and 50% PPE-HA-AgNP hydrogels were also determined to be significantly effective compared to the negative control group. As a result, the findings from this in vivo study provide evidence that AgNPs synthesized from PPE and incorporated into a CMC-HA hydrogel not only ensured infection control in an infected excision wound model but also significantly enhanced wound healing by supporting tissue repair processes in a multifaceted manner.

As an additional example, Permyakova et al. investigated the wound healing effect of curdlan–chitosan foams containing AgNPs (CUR/CS/Ag), on excision wounds in genetically type 2 diabetic db/db mice model [91]. Initially, full-thickness excision wounds measuring 1 cm × 1 cm were symmetrically created on the dorsal region of 5-month-old female db/db mice. Subsequently, different treatment groups were applied to the excision wounds created in diabetic mice: foam treatment containing only CUR/CS, AgNP-integrated CUR/CS/Ag foam treatment, and a negative control group that received no treatment. On the 24th day, wound healing was almost not observed in the negative control group, and in fact, the wound area increased by more than 20%. In contrast, about 20% wound closure was recorded in the group treated with CUR/CS foam, whereas complete healing (100% wound closure) was achieved in the group treated with CUR/CS/Ag foam. Furthermore, in the Phosphate-Buffered Saline (PBS) absorption test, which was used to simulate wound fluid, it was observed that the CUR/CS/Ag foam absorbed the fluid in 6 s, whereas the CUR/CS foam required 60 s. In conclusion, the findings from this in vivo study provide evidence that AgNPs incorporated into CUR/CS foam accelerated wound healing and promoted effective tissue repair in diabetic excision wounds.

As a further example, Yaqubi et al. investigated the wound healing effect of AgNPs synthesized from grape seed extract (GSE), which were stimulated by blue laser light, on infected incision wounds in female Wistar mice [92]. Initially, AgNPs were produced through a green synthesis method from GSE, and they were characterized as spherical with an average size of 80.24 ± 0.94 nm and an SPR peak at 434 nm. Subsequently, incision wounds measuring 2.5 cm in length and infected with *S. aureus* were created on the dorsal region of female Wistar mice. Different treatment groups were applied to the created infected incision wounds: a combination treatment of GSE-AgNPs and blue laser, blue laser treatment alone, GSE-AgNP treatment alone, and a negative control group with no treatment. According to the data obtained on day 5, the group treated with the combination of GSE-AgNPs and blue laser exhibited a 60% reduction in wound size and an 88.73% decrease in *S. aureus* bacterial load. Although the wound size reduction percentages for the other groups were not reported, minimal wound closure was observed in the negative control group, whereas moderate improvement was observed in the single-treatment groups treated with either blue laser or GSE-AgNPs alone. Ultimately, the findings from this in vivo study provide evidence that AgNPs obtained from GSE and stimulated by blue laser not only ensured infection control in the infected incision wound model but also significantly enhanced wound healing by supporting tissue repair processes in a multifaceted manner.

To summarize, numerous in vivo studies support that AgNPs accelerate the healing process and provide more effective and higher-quality healing in various wound models established in mice and rats. These findings are further supported by studies on wound models developed in mice or rats, including burn, infected burn, excision, infected excision, excision in diabetic mice and infected incision models as discussed in this section. On the other hand, in vivo studies investigating the effects of AgNPs are not limited to mice and rat models but are also conducted on different animal models.

#### 3.2.2. Rabbit Models

Rabbit models are another commonly used living organism in studies examining the effects of AgNPs on wound healing, even though less common than mice and rat models. In such in vivo studies, different wounds are created in rabbits under controlled conditions, treatment methods containing AgNPs are applied to these wounds and the healing process after treatment is analyzed.

Mukhtarovna et al. examined the wound healing effect of AgNPs produced from the *Aloe vera* plant via the green synthesis method on full-thickness excision wounds created on the ears of New Zealand white rabbits [93]. The produced AgNPs were determined to be spherical in shape and approximately 15 ± 3 nm in average size. Subsequently, three different treatment groups were administered to the excision wounds created in the ears of 15 NZW rabbits: a control group treated only with sterile saline solution (without AgNPs), an AgNP-low group treated with 0.1 mg/mL of the synthesized AgNP suspension, and an AgNP-high group treated with 1 mg/mL of the same suspension. To investigate whether the therapeutic effect of AgNPs is dose-dependent, they were administered at different concentrations. Sterile gauze dressings soaked in the respective treatment solutions were applied daily to the excision wounds in rabbits for 14 days and secured with adhesive bandages. At the end of day 14, according to the analysis, a wound closure rate of 95.2% ± 2.1 was measured in the AgNP-high group, whereas 88.7% ± 3.5 was recorded in the AgNP-low group and 76.4% ± 4.2 in the negative control group. In addition, superior healing quality in the AgNP-high group was confirmed by histological analysis, with re-epithelialization, collagen accumulation, and neovascularization scores of 2.8 ± 0.4, 2.6 ± 0.5, and 2.4 ± 0.5, respectively. Taken together, the findings from this in vivo study provide evidence that AgNPs enhance wound healing outcomes in a dose-dependent manner by accelerating wound closure and promoting effective tissue repair.

Almohamad et al. investigated the wound healing effects of the Acticoat^®^ dressing on surgically created cecal anastomosis in NZW rabbits. Acticoat^®^ was described as an alginate-based dressing directly coated with 15 nm AgNPs through the physical vapor deposition (PVD) method [94]. In the initial phase, the cecum (the section located at the beginning of the large intestine) of 48 healthy male NZW rabbits was incised in a full-thickness manner. Following this, an end-to-end anastomosis was performed, in which two surgically transected bowel segments were joined by suturing their ends. The rabbits after these procedures were divided into two groups: in the control group, only 3-0 polydioxanone sutures were used for anastomosis, whereas in the AgNP group, a 2 × 5 cm Acticoat^®^ dressing layer was placed over the suture line. Subsequently, various tests and analyses were conducted on days 7, 15, and 30 in the rabbits to evaluate the wound healing process, including measurement of the cecal diameter at the anastomosis line to assess possible narrowing (stenosis). The control group showed values of 6.5 ± 0.23 mm, 9.1 ± 0.15 mm and 10.8 ± 0.3 mm on days 7, 15 and 30, respectively, while the AgNP group showed values of 20.9 ± 0.6 mm, 22.6 ± 0.41 mm and 27.5 ± 0.82 mm. To evaluate pain levels in the rabbits, the Bristol Rabbit Pain Score (BRPS) was analyzed, and the AgNP group exhibited significantly lower pain scores compared to the control group. Macroscopic examinations were performed to evaluate the presence of leakage or adhesions at the anastomosis line, and while no anastomotic leakage was observed in any of the rabbits in the AgNP group (100%), only 20.8% of the rabbits in the control group exhibited an intact anastomotic line. Additionally, the percentage of rabbits with an adhesion score of 0 was measured as 70.9% in the AgNP group, whereas this percentage was only 8.3% in the control group. To determine the mechanical strength of the anastomotic region, bursting pressure and tensile strength tests were performed; in the AgNP group, both parameters showed significantly higher values compared to the control group on days 7, 15, and 30. In addition, histopathological analyses demonstrated that the anastomotic line treated with AgNPs exhibited significantly lower levels of inflammation and markedly higher collagen accumulation. Ultimately, the findings from this in vivo study clearly demonstrated that AgNP-containing Acticoat^®^ dressing layer accelerated wound healing following surgical anastomosis, reduced postoperative pain, decreased long-term complications such as adhesions and leakages, enhanced mechanical strength, preserved structural tissue integrity, and reduced inflammation. In summary, this in vivo study has demonstrated the beneficial contribution of AgNPs in surgical treatments.

As another example, Sadan et al. examined the effect of a hydrogel incorporating AgNPs produced via a green synthesis method from the plant *Trigonella foenum-graecum* (FG), on the healing of a bone defect model created in the tibia of NZW rabbits [95]. The prepared AgNPs-FG were determined to be spherical in shape, with an average size of approximately 16.73 ± 3.68 nm and a zeta potential of −7.8 ± 0.518 mV. Subsequently, four different treatment groups were applied to bone defects created in the tibiae of 40 NZW rabbits: the control group was treated only with sterile saline solution; one group was treated only with hydrogel, another group was treated with hydrogel containing FG, and the last group was treated with hydrogel containing AgNPs-FG. To evaluate the extent of bone gap closure, radiographs were regularly taken of the rabbits, and in the AgNPs-FG group, complete integration was observed at week 8 with a perfect Radiographic Union Scale (RUS) score of 10/10. In contrast, a score of 3 was recorded in both the control and only hydrogel groups, while in the FG hydrogel group, scores between 4 and 10 were recorded. To investigate tissue regeneration at the biochemical level, the levels of Bone Alkaline Phosphatase (BAP) and Osteocalcin (OC), which indicate the activity of osteoblasts, the cells responsible for bone formation, were measured. In the AgNPs-FG group, a significant increase in BAP levels was observed from week 2 and was maintained until week 8. In the same group, an increase in osteocalcin (OC) levels was observed between weeks 2 and 6, followed by a return to normal by week 8. On the other hand, in the FG-hydrogel group, an increase in OC levels was detected only at weeks 2 and 8, whereas no significant changes were recorded in the group containing only hydrogel and in the control group. Furthermore, in order to analyze the mineralization process, calcium and phosphorus levels were measured. In the AgNPs-FG group, a significant increase in calcium levels was measured between weeks 2 and 8, while a significant rise in phosphorus levels was noted between weeks 6 and 8. In addition, a significantly lower level of inflammation was revealed by histopathological analyses in the group treated with AgNPs-FG. In conclusion, the findings from this in vivo study demonstrated that hydrogel treatment containing AgNPs FG preserved tissue integrity following bone defect, supported new bone formation by enhancing osteoblast activity and mineralization, reduced inflammation, and promoted high-quality bone regeneration.

In another example, Al Moghazy et al. investigated the healing effect of resin composites incorporating AgNPs on wounds generated by revealing the pulp (the living tissue located in the innermost part of the tooth, consisting of blood vessels and nerves) in the teeth of NZW rabbits [96]. The generated AgNPs were determined to be spherical in shape and approximately 50 nm. Subsequently, three different treatments were applied to the wounds formed by revealing the pulp in the teeth of the 18 NZW rabbits. In the negative control group, the pulp was coated only with resin composite; in the positive control group, the pulp was coated with Mineral Trioxide Aggregate (MTA) and resin composite was placed on top; in the experimental group, the pulp was coated with a bonding agent modified with AgNPs, and resin composite was then applied over it. At the end of four weeks, based on histological examinations, severe inflammation was observed in the control group at a rate of 43.93% ± 1.87, along with necrosis and a thin and discontinuous newly formed bone-like dentin layer covering the pulp; whereas in the MTA group, a lower inflammation rate of 20.12% ± 1.20 and the formation of a thin and discontinuous dentin layer were reported. In the AgNP group, the inflammation rate was found to be significantly lower (14.7% ± 1.07), no necrosis was observed, and a thick and continuous dentin layer was formed in all samples. In other words, the AgNPs completely coated the pulp like a wall. Taken together, the findings of this in vivo experiment provided strong evidence that AgNPs not only reduce bacterial contamination but also accelerate the healing process by supporting regeneration in the pulp tissue.

In a further example, Masood et al. investigated the effect of a chitosan-PEG-based CH/PEG/Ag hydrogel incorporated with AgNPs on excisional wounds created in alloxan-induced diabetic rabbits [97]. During this hydrogel synthesis, chitosan contributed through its wound healing and antibacterial properties, while PEG ensured the controlled release of AgNPs and its structural stability. In SEM analysis, not only the presence of AgNPs with a size of 99.1 ± 2.3 nm was confirmed, but also the presence of pores with a diameter of 130–140 nm within the hydrogel structure was determined. These pores contribute to wound healing by both absorbing wound fluid and supporting moisture balance, gas permeability, and cell adhesion. Additionally, the CH/PEG/Ag hydrogel was evaluated for both its antibacterial and antioxidant activities through in vitro tests. According to colony count analysis, the hydrogel exhibited strong antibacterial activity by providing 99.7% ± 3.1 and 99.2% ± 1.9 bacterial inhibition against *E. coli* and *S. aureus*, respectively; similarly according to the DPPH test, the hydrogel also exhibited strong antioxidant activity with a radical scavenging capacity of up to 85%. Subsequently, the wound healing effect of this CH/PEG/Ag hydrogel was evaluated by creating full-thickness excisional wounds, each measuring 20 mm^2^, on the dorsal region of NZW rabbits induced with diabetes through alloxan monohydrate (a diabetes-inducing agent). Five treatment groups were established for the wounded rabbits: a negative control group (no treatment), a positive control group (treatment with 0.2% nitrofurazone), treatment with AgNP solution alone, treatment with blank CH/PEG hydrogel (without AgNPs), and treatment with CH/PEG hydrogel loaded with AgNPs. By day 4, the CH/PEG/Ag hydrogel group exhibited the highest wound closure rate (47.7% ± 1.8), whereas the positive control, AgNP, CH/PEG, and negative control groups showed closure rates of 32.9% ± 2.1, 26.9% ± 1.4, 43.5% ± 2.2, and 12.6% ± 1.3, respectively. As a result, the findings obtained from this in vivo experiment clearly demonstrated that the AgNP-loaded chitosan-PEG hydrogel significantly accelerated wound healing and simultaneously promoted high-quality tissue regeneration in an excisional wound model established in diabetic rabbits.

To summarize, it has been evaluated that AgNPs can accelerate the healing process and promote more effective and higher-quality healing in various wound environments created in rabbits. In in vivo studies using rabbits as the animal model, various wound models have been successfully established, including multiple excisional wounds on the ears, an anastomosis in the cecal region, a defect in the tibial bone, and pulp exposure in the tooth. These various wound environments have been established possible due to the anatomical structure of rabbits resembling human skin, the ease of creating controlled wounds, high measurement precision, and their immunological similarity to human biology. On the other hand, in vivo studies have not been limited to this animal model.

#### 3.2.3. Porcine Model

Porcine models are rarely used living organisms in studies examining the effects of AgNPs on wound healing. In such in vivo studies, the burn wound model is commonly preferred owing to the porcine skin exhibiting thermal responses similar to those of human skin, along with the model’s repeatability and greater ethical acceptability.

Yang et al. developed a sandwich-structured wound dressing containing AgNPs and investigated its healing effect on severe burn wounds created on a porcine model [98]. Initially conducted in vitro tests confirmed that the developed nanocomposite PU-CSNWF/AgNPs-CS/COL sandwich wound dressing exhibits significant antibacterial activity and high cellular biocompatibility. According to the shake flask test, the sandwich wound dressing achieved antibacterial inhibition rates of 98.55%, 97.36%, and 98.21% against *E. coli*, *P. aeruginosa*, and *S. aureus*, respectively, while the live/dead cell staining test revealed that the majority of fibroblast cells remained viable. Only a polyurethane membrane covered with sterile cotton gauze (PU-Gauze) in the negative control group; silver sulfadiazine cream covered with a polyurethane membrane (PU-Gauze-SSD) in the positive control group; and, respectively, chitosan nonwoven fabric (PU-CSNWF), AgNP-loaded chitosan fabric (PU-CSNWF/AgNPs), collagen–chitosan sponge (PU-CS/COL sponge), and the PU-CSNWF/AgNPs-CS/COL wound dressing in the other groups were applied. On day 17, a wound closure rate of 73.9% ± 4.05 was observed in the positive control group (PU-Gauze-SSD), and 77.6% ± 3.88 in the PU-CS/COL group treated with only collagen–chitosan sponge, whereas in the other three groups (PU-Gauze, PU-CSNWF, PU-CSNWF/AgNPs), more than 85% of the wound area remained unhealed. In contrast, the PU-CSNWF/AgNPs-CS/COL group showed superior results compared to all other groups in terms of wound closure, with a healing rate of 49.8% ± 9.85, and achieved complete, smooth and without scarring full-thickness re-epithelialization by day 28. Moreover, histological examinations also confirmed the most prominent epidermal regeneration, collagen remodeling, and minimal inflammatory infiltration in this group. Taken together, the findings demonstrate that AgNP-loaded multilayer wound dressing not only provides antibacterial activity, but can also accelerate angiogenesis, re-epithelialization, and tissue regeneration by reducing the inflammatory response, thereby promoting rapid and high-quality healing without scar formation in deep dermal burn wounds in porcine.

In another example, Do et al. evaluated the effect of a gelatin-AgNP loaded polycaprolactone (PCL) based wound dressing (PCLGelAg) on the healing process of partial-thickness thermal burn wounds created on a porcine model [99]. The produced PCLGelAg was subjected to comparison with two commercial silver-containing products, Aquacel^®^ Ag and UrgoTul^®^ SSD. In order to compare their antibacterial activities, agar diffusion, MIC/MBC, and time-kill tests were performed in vitro against *P. aeruginosa* and *S. aureus*, in which PCLGelAg demonstrated remarkable performance, with an MBC value of 2.44 µg/mL and successfully killed 99.99% of the bacteria within 12 h. In the following step, different treatment methods were applied to the 24 burn regions generated on the pigs. Only sterile gauze was applied to the negative control group, while the commercial wound dressings Aquacel^®^ Ag and UrgoTul^®^ SSD were applied to the positive control groups, respectively, and the PCLGelAg wound dressing was applied to the experimental group. On day 20, the mean wound areas were measured to be 3.1 cm^2^ in the negative control group, 2.7 cm^2^ in the PCLGelAg group, 2.4 cm^2^ in the Aquacel^®^ Ag group, and 2.3 cm^2^ in the UrgoTul^®^ SSD group. Based on these values, the calculated wound closure rates were 29.5%, 38.0%, 42.8%, and 45.2%, respectively. The commercial products provided a significant level of wound closure, while PCLGelAg demonstrated a comparable efficacy to the commercial products and markedly greater efficacy than the negative control group. In addition, histopathological examinations demonstrated that the Aquacel^®^ Ag and UrgoTul^®^ SSD groups exhibited full-thickness epidermal regeneration, minimal inflammatory cell infiltration, and dense collagen accumulation, while no signs of necrosis were observed. In the PCLGelAg group, full-thickness epidermal regeneration was observed; however, compared to the commercial groups, inflammatory cell infiltration was higher, and collagen accumulation was lower. In conclusion, these findings highlight that the gelatin-AgNP loaded PCL-based wound dressing led to a moderate but distinct improvement compared to commercial products, such as wound closure rate, level of inflammation, epidermal remodeling, and collagen accumulation.

As a further example, Ross et al. comparatively examined the effectiveness of three different commercially available silver-based wound dressings, which had previously been evaluated in vitro, this time in infected burn wounds established on porcine models [100]. The dorsal regions of three pigs were infected with a mixture of *S. aureus* (USA300) and *P. aeruginosa* (ATCC 27853) at a volume of 0.5 mL and a concentration of 10^8^ CFU/mL. Only sterile gauze was applied to the negative control group, while the experimental groups were treated with the commercial wound dressings: silver-coated nylon (Silverlon^®^), highly oxidized silver salts (Exsalt SD7), and nanocrystalline silver (Acticoat^®^ Flex 3), respectively. According to the analyses performed on day 1, the average total bacterial load in the negative control group was measured as 5.95 log_10_ CFU/g, whereas in the Acticoat^®^ Flex 3 group containing nanocrystalline silver, this value significantly decreased to 1.93 log_10_ CFU/g. Simultaneously, bacterial load reductions of 4.04 and 4.97 log_10_ were observed for *S. aureus* and *P. aeruginosa*, respectively, further highlighting the effectiveness of the nanocrystalline silver group. While reductions in bacterial load were noted in the silver-coated nylon (Silverlon^®^) and highly oxidized silver salt (Exsalt^®^ SD7) groups (estimated at 0.5–3 log_10_), these changes were not found to be statistically significant. Furthermore, histopathological examinations reported that no group showed a distinct advantage in terms of edema and erythema (redness) levels, and no statistically significant wound closure was shown in any group based on re-epithelialization rates. In summary, after burn wounds were generated, severe tissue damage extending into the deep layers of the dermis was identified in all groups; however, no microscopically significant difference in healing was observed between the treatment groups after 3 days. Ultimately, the nanocrystalline silver-containing wound dressing provided a stronger antibacterial effect in infected burns by significantly reducing the total bacterial load within the first 24 h compared to other commercial silver-based products; however, it showed no significant advantage in wound healing among the treatment groups.

To summarize, as demonstrated in a very limited number of in vivo studies, AgNP-based treatments support effective and high-quality wound healing in porcine burn models. The evaluation of AgNP-based treatments in porcine models, which represent the in vivo model most similar to human biology, is of great importance for increasing the reliability of preclinical studies and strengthening the translatability of the results to humans. Nevertheless, owing to the high cost of pigs as well as ethical and logistical challenges, it is observed that they are used in the literature to a limited extent compared to other animal models.

Taken together, as observed in the example studies presented above, in vivo experiments conducted in mice, rats, rabbits, and porcine models have highlighted the potential effects of AgNPs across various wound types. Nevertheless, the effectiveness and safety of these findings can only be confirmed through well-designed clinical studies.

### 3.3. Clinical Applications in Human Wounds

Clinical studies conducted on human subjects under real-life conditions are of great importance for evaluating the validity of in vitro and in vivo findings on the effects of AgNPs on wound healing. Through these studies, the benefits provided by AgNPs in different wound models, the risks in terms of patient safety, and the long-term outcomes are comprehensively evaluated. In short, this process represents one of the fundamental steps that enables the transformation of scientific evidence into medical practice.

Hurd et al. examined the effects of the nanocrystalline silver (NCS)-coated ACTICOAT™ Flex 7 wound dressing, which is used within the integrated care bundle (ICB), on diverse open chronic wounds that occurred due to different causes [101]. A total of 2572 patients with pressure ulcers, venous leg ulcers, open surgical incisions, burn wounds, or diabetic foot ulcers, which are chronic wounds of different stages and sizes, without active infection, were included in this clinical study. In many of the patients involved in the study, additional health problems such as diabetes, cardiac diseases, and renal disorders were present. The patients were divided into two groups: an ICB containing NCS dressings was applied to 330 patients, while standard treatment without silver was applied to 2242 patients. Older patients and those with additional health problems were more frequently observed in the group treated with ICB + NCS. Several parameters, including healing rate, dressing frequency, systemic infection rate, the Bates-Jensen Wound Assessment Tool (BWAT) score (https://www.sralab.org/rehabilitation-measures/bates-jensen-wound-assessment-tool, accessed on 10 September 2025), and nursing service cost, were evaluated during the treatment period. The ICB + NCS dressing was applied for 10.46 weeks, and was changed on average every 3.98 days. In contrast, the standard dressing was applied for an average of 25.49 weeks, and was required to be changed every 1.87 days. Additionally, systemic infection rates were determined as 0.9% in the ICB + NCS group and 3.1% in the standard treatment group. Furthermore, the mean BWAT score was recorded as 31.4 in the ICB + NCS group, whereas it was 33.2 in the standard treatment group. Ultimately, all the findings obtained from this clinical study demonstrated that the NCS coated wound dressing used within the ICB accelerated wound healing, reduced dressing change frequency, lowered systemic infection rates, and decreased total care costs, even among older individuals with several additional health problems.

In another clinical study, Asgari et al. evaluated the effects of an AgNP-containing dressing and a hydrocolloid dressing on the healing process of stage II pressure ulcers (PUs) in patients with spinal cord injury (SCI) [102]. A total of 70 patients aged between 18 and 65 years, who had stage II pressure ulcers (PUs) without infection or necrotic tissue, most of which were irregular in shape, were included in this study. These 70 patients were randomly divided into two groups: one group was treated with a wound dressing incorporating AgNPs at 1.7 ppm/cm^2^, while a hydrocolloid dressing was applied to the other group. Both wound dressings, 5 × 5 cm or 10 × 10 cm in size, were applied for a period of 12 days, and were required to be changed every 3 days. Therefore, analyses conducted at four separate time points were performed using the BWAT, a wound assessment system based on 13 parameters in which better healing is indicated by lower scores. According to the findings, the BWAT score in the group treated with the AgNP-containing dressing was observed to be 27.25 ± 6.7 at the beginning and was reduced to 24.3 ± 7.1 by the end of the 12-day period. Similarly, in the group treated with the hydrocolloid dressing, the BWAT score was observed to be 23.87 ± 4.09 at the beginning and was recorded as 22.80 ± 5.3 by the end of the 12-day period. However, no statistically significant difference was observed between the two wound dressings. This situation is thought to be associated with several factors, including the low concentration of AgNPs used, the fact that the study was conducted on wounds without infection or necrosis, and the ability of both dressings to maintain a moist healing environment at the wound area. In conclusion, the findings of this clinical study demonstrated that wound dressings containing AgNPs are a safe and effective option for the treatment of PUs and provide healing results comparable to those of hydrocolloid wound dressings in non-infected wounds.

As another example, Zhang et al. retrospectively examined the effects of a thermoplastic polyurethane (TPU) wound dressing coated with AgNPs on postoperative wounds in diabetic patients with open fractures of the lower extremities [103]. A total of 98 diabetic patients with moderate (Gustilo type II) or severe but surgically closable (Gustilo type IIIA) open fractures, with a mean age of 56.22 years, were included in this study. These patients were divided into two groups: 41 patients were treated with AgNP/TPU wound dressings, while 57 patients were administered standard wound dressings (benzalkonium chloride or iodophor). The AgNP/TPU wound dressings applied in the observation group were coated with nanospherical AgNPs, approximately 95 nm in diameter, which were uniformly loaded onto the TPU membrane. During the treatment period, several parameters were evaluated, including the level of inflammation, rate of bacterial infection, wound healing time, frequency of dressing changes, pain level (VAS score), functional recovery (FIM score), and quality of life (SF-36). After 7 days of treatment, the white blood cell (WBC) level in the AgNP/TPU group was measured as 6.21 ± 1.04, the C-reactive protein (CRP) level as 12.84 ± 4.91 mg/L, the IL-6 level as 19.52 ± 3.67 pg/mL, and the TNF-α level as 11.78 ± 2.16 pg/mL; whereas in the standard treatment group, these values were measured as 8.34 ± 1.65, 19.12 ± 5.43 mg/L, 27.13 ± 4.25 pg/mL, and 17.03 ± 2.99 pg/mL, respectively. The infection rate in the wound area was found to have decreased to 4.88% in the AgNP/TPU treatment group; whereas in the standard treatment group, this rate was recorded as 12.28%. Additionally, in the AgNP/TPU treatment group, complete wound healing was observed after 13.47 ± 2.14 days, and an average of 6.52 ± 1.32 dressing changes were performed during this period. In contrast, in the standard treatment group, complete wound healing was observed after 18.03 ± 3.01 days, during which an average of 9.11 ± 1.75 dressing changes was required. In patients treated with AgNP/TPU, VAS scores were measured as 2.21 ± 0.87 and FIM scores as 113.26 ± 7.52, while in patients treated with standard treatment, these scores were measured as 4.09 ± 1.03 and 97.45 ± 8.68, respectively. In addition, the total complication proportion (including complications such as slow wound healing, wound infection, scar formation, soft tissue necrosis, and chronic pain) was recorded as only 17.07% in the AgNP/TPU group, whereas this proportion was recorded as 35.09% in the group treated with standard treatment. As a result, the findings obtained from this clinical study demonstrated that the TPU wound dressing coated with AgNP accelerates wound healing by reducing the risk of infection, reduces pain, lowers complication proportions, and remains effective even in the presence of systemic diseases such as diabetes.

In another example, Budini et al. investigated the effects of an AgNP-based wound dressing composed of a TLC-Ag colloidal lipid matrix combined with polyacrylic absorbent fibers, on complex chronic wounds of different origins [104]. In this clinical study, 10 patients with various chronic wounds that had remained unhealed for approximately 3 to 40 weeks, ranging in size from 1 to 30 cm^2^ and not infected but at risk of infection, were included. The AgNP-based wound dressing, which could exhibit both antibacterial and absorptive properties owing to its TLC-Ag matrix and polyacrylic fibers, was applied to all patients every 3 to 5 days for about 9.6 weeks. The control group was not included in this study. Following the treatment, the wound area, which was initially 20 ± 9.3 cm^2^ on average, was reduced to 11.3 ± 8.4 cm^2^, while the wound depth was reduced from an average of 2.6 mm to 0.1 mm. Additionally, the initially observed 7% necrotic tissue in the wound bed was completely eliminated. The amount of fibrin (dead tissue covering) was reduced from 50% to 22%, while the percentages of granulation tissue and re-epithelialization, initially measured at 26% and 16%, respectively, were increased to 40% and 39%. Meanwhile, the number of patients with redness around the wound site was reduced from 6 to 1, local heat and edema were completely resolved, and the number of patients with pain reported during dressing changes was reduced from 5 to 3. On the other hand, 5 patients were indicated for surgery at the end of treatment, one of them was treated with primary suture and four of them were treated with thin dermo-epidermal graft surgery. In conclusion, AgNP-based wound dressing provided only a limited reduction in total wound area, but was markedly effective in wound bed debridement, supporting tissue quality, and preparing the wound suitable for surgical intervention.

As a further example, Adarsh et al. examined the effects of a nanogel wound dressing incorporating AgNPs on diabetic foot ulcer (DFU) wounds [105]. A total of 60 patients with stage II diabetic foot ulcers, aged between 18 and 75 years, were included in this clinical study. These patients were randomly divided into two groups: 30 patients were treated with AgNP-incorporated nanogel, while the other 30 patients were administered standard saline dressing. These two dressings were applied to the wounds every other day under aseptic conditions, and when required, debridement (the removal of necrotic or infected tissue) was performed. During the treatment period, parameters were assessed, including wound size, granulation tissue formation, and the presence of slough. In the 4th week of the treatment process, the wound size in the group treated with AgNP-containing nanogel was measured as 26.1 ± 30.5 cm^2^, and as 9.2 ± 20.2 cm^2^ in the 8th week. In contrast, in the control group, these values were recorded as 51.6 ± 26.1 cm^2^ at week 4 and 19.6 ± 18.6 cm^2^ at week 8. As for granulation tissue, at the 8th week, healthy granulation development was observed in 96.7% of the patients in the nanogel group, whereas this rate was 76.7% in the control group. Additionally, at the end of the 8th week, the presence of slough in the wound area was identified in 16.7% of the patients in the nanogel group and in 46.7% of the patients in the control group, respectively. Overall, the findings of this clinical study demonstrated the potential of AgNP-incorporating nanogel dressing in the treatment of diabetic foot ulcers by accelerating wound size reduction, promoting tissue regeneration, and reducing slough.

To summarize, AgNPs exhibit the potential for safe and effective use under real-life conditions. They offer valuable findings for clinical applications across diverse patient groups and wound types. Nevertheless, in order for these findings to become applicable, additional comprehensive clinical studies are required.

Taken all together, the above examples of in vitro, in vivo, and clinical studies support the positive effects of AgNPs on wound healing. These studies have demonstrated that AgNPs contribute to positive results in wound healing processes through their strong antibacterial properties, as well as their anti-inflammatory, antioxidant, and cell proliferation-promoting properties. These positive effects observed in studies conducted on different types of wound models have confirmed that AgNPs both significantly accelerate wound healing and support tissue repair processes in a multifaceted manner. To complement this discussion, Table 2 provides a categorized summary of further representative in vivo and clinical studies, with particular emphasis on recent studies published after 2020. Nevertheless, the current studies within this discipline remain insufficient in terms of long-term safety. In particular, the uncertainties regarding toxicity represent one of the major challenges to the routine clinical use of AgNPs.

## 4. Safety, Cytotoxicity, and Biocompatibility of AgNPs

As discussed in the previous sections, AgNPs support wound healing processes in a multifaceted manner by enabling the inhibition of infections, the regulation of inflammatory responses, the reduction in oxidative stress, and the acceleration of tissue regeneration due to their antibacterial, anti-inflammatory, antioxidant, and cell proliferation-promoting properties. Nevertheless, many uncertainties still persist concerning the potential risks of exposure to AgNP-based products, specifically their short- and long-term toxic effects on living organisms and ecosystems [51]. The toxicity of AgNPs is influenced by several physicochemical and biological parameters, including particle size and shape, surface features, concentration, ion release capacity, oxidative stress potential, agglomeration tendency, and route of administration [123]. These parameters are fundamental to the toxicity of AgNPs.

Many studies have demonstrated that AgNPs exhibit increased toxicity when used at high concentrations [124,125]. Despite their multifunctional biological properties, AgNPs may negatively affect wound healing when used at high concentrations by increasing ROS generation, inhibiting cell proliferation, prolonging inflammation, and inducing apoptosis [126]. Exposure to high concentrations of AgNPs increases the intracellular release of Ag^+^ ions, which can disrupt mitochondrial function, leading to the accumulation of ROS and increasing oxidative stress levels. The resulting oxidative stress causes damage to lipids, proteins, and DNA, initiating apoptotic signals in intracellular structures and leading to the loss of cells responsible for wound healing [127]. The loss or inhibition of the proliferation of cells fundamental to wound repair, such as fibroblasts and keratinocytes, impair new tissue formation and delays wound closure [128]. Moreover, exposure to high AgNP concentrations can cause prolonged activation of inflammatory cells and increased cytokine production, which may lead to the development of chronic wounds [123].

In addition, some studies in the literature have demonstrated the effects of the physicochemical properties of AgNPs, such as particle size, shape, and surface features, on their toxicity [129,130,131,132]. For example, a study conducted by Cunningham et al. confirmed that the size of AgNPs directly affects their toxicity [133]. AgNPs stabilized with a hybrid lipid coating that inhibits Ag^+^ ion release, in sizes of 20, 40, 60, 80, and 100 nm, were administered to zebrafish embryos at doses ranging from 0.25 to 12 mg/L. Complete mortality was caused by the 20 nm AgNPs at 12 mg/L, and severe structural abnormalities were induced at concentrations as low as 3 mg/L; whereas no toxicity was observed with the 80 and 100 nm AgNPs. It was shown by ICP-MS analyses that all sizes of AgNPs were taken up by zebrafish embryos, whereas significantly higher accumulation levels were exhibited by smaller NPs. These findings indicate that the toxicity of AgNPs increases as particle size decreases, independent of Ag^+^ ion release. As another example, a study conducted by Harper et al. indicated that the shape and surface features of AgNPs directly affect their toxicity [134]. Five different surface coatings were applied to spherical (AgNS) and triangular plate-shaped (AgNPL) NPs: citrate (Cit), sodium oleate-phosphatidylcholine (SOA-PC), short-chain thiol (SOA-PC-PT), long-chain thiol (SOA-PC-HT), and additionally purified (SOA-PC-HT(P)). Subsequently, they were stabilized to prevent Ag^+^ release. These prepared AgNPs were tested at concentrations of 0.1 and 0.5 mg/L on multiple organism models containing bacteria (*E. coli*), algae (*Chlamydomonas reinhardtii*), water fleas (*Daphnia magna*), and zebrafish embryos (*Danio rerio*). When compared in terms of shape, up to 100% mortality was observed to be caused by Cit- and SOA-PC-coated AgNSs, whereas minimal or no toxicity was observed to be exhibited by AgNPLs under the same conditions. The ability of AgNSs, owing to their smaller and spherical structures, to more easily interact with cell membranes, which facilitates greater uptake into organisms, can explain this difference. When compared in terms of surface features, AgNPs with complex coatings (SOA-PC-HT and HT(P)) were observed to exhibit less toxicity in organisms than AgNPs with simple coatings (Cit, SOA-PC). The ability of complex coatings to stabilize the surface of AgNPs can explain this difference, thereby reducing direct interactions with cell membranes and limiting their uptake into organisms. Moreover, some studies indicate that various surface coatings applied to AgNPs alter their surface charge, thereby affecting their toxicity in cells [135,136].

Several studies in the literature have shown that AgNP synthesis methods markedly affect these parameters underlying toxicity. These effects mainly arise from variations introduced during the synthesis process [137,138]. The physical, chemical, and green synthesis methods used to produce AgNPs can influence their in vitro toxicity levels, in vivo biocompatibility profile, and environmental risks [139].

The physical synthesis methods usually include costly processes that require advanced technology and energy, such as evaporation–condensation and laser ablation. This synthesis process does not require chemical reagents or toxic solvents. However, AgNPs produced by this method generally lack surface coatings and have irregular shapes, which may lead to uncontrolled Ag^+^ ion release and damage to the cell membrane. Overall, physical synthesis methods, despite generating no toxic waste, have limited applicability owing to their unpredictable effects on biological systems and costly processes [140]. Another synthesis method, chemical synthesis, usually requires complex laboratory procedures and involves the use of toxic solvents, chemical reagents, and stabilizers. Therefore, AgNPs produced by this method may contain non-biodegradable residues or toxic by-products; these can lead to unwanted cell damage. Moreover, this synthesis method is costly due to high temperature and energy requirements, and it also causes environmental pollution by generating large amounts of chemical waste [141].

On the other hand, green synthesis methods are based on simple processes carried out under low temperature and energy conditions, using biomolecules derived from plant extracts, biopolymers, or other natural resources [15]. These biomolecules act both as reducing agents and as natural coatings on the nanoparticle surface, thereby enhancing particle stability, preventing agglomeration, and enabling the controlled release of Ag^+^ ions. Antioxidant compounds present in plant extracts, such as flavonoids and polyphenols, provide stabilization while also imparting additional antioxidant and anti-inflammatory properties that may support wound healing. In this way, lower cytotoxicity and higher biocompatibility may be obtained compared with uncoated, irregularly shaped particles synthesized by conventional methods [67].

In addition to plant extracts, biosynthesis approaches have been developed in some studies in which microorganisms such as bacteria, fungi, and algae are directly employed. In these systems, enzymes and metabolites secreted by microorganisms direct nanoparticle nucleation and growth, allowing for narrow size distribution and more homogeneous structures. Moreover, secreted proteins and polysaccharides serve as natural capping agents, improving biocompatibility and broadening surface characteristics. Certain proteins and biopolymers secreted by microorganisms may also exhibit inherent antibacterial activity, which can further contribute to infection control at the wound site. This enables more precise control over nanoparticle morphology and surface chemistry [54].

In conclusion, green synthesis methods offer a safer and more sustainable alternative to conventional approaches, owing both to the environmentally friendly and economical nature of their production process and to the more stable, biocompatible, and low-toxicity profiles of the resulting AgNPs. Furthermore, their low-cost and easily scalable nature provides notable advantages for applicability in both laboratory settings and industrial-scale production. In terms of environmental impact, the minimization of toxic waste generation and the use of renewable biological resources make this method an attractive option for sustainable nanotechnology applications [137].

In a study conducted by Zhang et al., the antibacterial properties and phytotoxicity of AgNPs produced via the green synthesis method using plant-based cucumber leaf extract (gc-AgNPs) and rice husk extract (gr-AgNPs) were comparatively evaluated against AgNPs produced via the chemical synthesis method (chem-AgNPs) [142]. When compared in terms of antibacterial activity, 32.5% ± 5.7 growth inhibition against E. coli was exhibited by chem-AgNPs, whereas a stronger and more prolonged effect, with 42.6% ± 1.3 inhibition, was exhibited by gr-AgNPs. When compared in terms of phytotoxic effects, serious oxidative stress in cucumber plants was caused by chem-AgNPs, with approximately a 3.7-fold increase in MDA levels; carotenoid pigment and globulin protein levels were also significantly decreased. In contrast, almost no phytotoxic effect was exhibited by AgNPs produced via green synthesis by positively influencing photosynthetic pigments and mineral balance, and metabolic benefits were even provided.

In addition, various in vitro and in vivo studies have demonstrated that AgNPs produced via green synthesis methods exhibit lower cytotoxicity in cell lines, resulting in less inhibition of cellular processes involved in wound healing. For instance, in a study conducted by Sharma et al., AgNPs produced via the green synthesis method using the *Cyperus rotundus* plant were incorporated into a Carbopol-based hydrogel system for wound treatment, and their biological activities were evaluated in a multifaceted manner [143]. Initially, the AgNP-hydrogel produced via the green synthesis method was determined to exhibit a marked antibacterial effect against *E. coli* and *S. aureus*. Subsequently, the wound healing potential of the AgNP-hydrogel was demonstrated due to its antioxidant activity (IC_50_ = 76.56 µg/mL) and anti-inflammatory activity (74.82% inhibition). In cytotoxicity analyses, 98% cell viability in HEK293 cells was shown by the AgNP-hydrogel, thereby demonstrating its high biocompatibility. Moreover, complete wound closure was observed in the in vitro scratch assay, and 86% re-epithelialization was achieved in the in vivo rat model. All these findings indicate that AgNPs synthesized via the green synthesis method could provide a safe and effective treatment alternative.

To summarize, although AgNPs provide significant wound healing effects through their multifaceted properties when used in wound treatments, their toxicological effects remain a critical concern. Especially in wound healing applications, the functionality of AgNPs should be evaluated by considering both their healing capacity and their potential toxic properties. The potential toxicity of AgNPs depends on both their physicochemical and biological parameters. While physicochemical properties such as particle size, shape, surface characteristics, ion release capacity, and agglomeration tendency may vary depending on the synthesis methods, oxidative stress potential, route of administration, and concentration (dose/exposure) are considered biological parameters. According to the literature, AgNPs produced through green synthesis methods are generally more biocompatible and exhibit minimal toxicity. Therefore, in the development of wound healing treatments, these parameters should be considered, and AgNPs obtained through the green synthesis method should be preferred. In addition, comprehensive cytotoxicity tests must be performed before their application.

## 5. Conclusions & Future Perspective

AgNPs have become a significant subject of research, particularly in the field of wound healing, owing to their multifunctional biological properties. Their antibacterial properties contribute to the inhibition of infections, anti-inflammatory properties support the regulation of inflammation, and antioxidant properties facilitate the reduction in oxidative stress, while their cell proliferation-promoting properties accelerate tissue regeneration processes. Moreover, AgNPs can exhibit synergistic effects with antibiotics against resistant bacteria, thereby enhancing the efficacy of antibiotics or restoring their lost effectiveness. These simultaneous biological effects have established AgNPs as a remarkable therapeutic agent for wound healing applications. It has been clearly demonstrated in numerous in vitro, in vivo, and clinical studies presented in this review that AgNPs, either monotherapy or incorporated into composite structures in various formulations such as nano-gels, hydrogels, powders, ointments, and sprays, provide significant benefits in a wide range of wound types, including burns, excisional and incisional wounds, surgical wounds, and diabetic ulcers. However, despite these significant benefits of AgNPs, the existing studies in the literature also present several important limitations.

One of these limitations is the insufficient use of porcine models in in vivo studies, despite their significant potential for evaluating wound healing due to their close structural and physiological similarity to human skin. The majority of studies are still conducted on animal models such as mice and rats, while comparatively fewer studies are conducted on rabbit models. This limitation complicates the effective translation of experimental data into clinical reality. Therefore, the use of porcine and rabbit models should be increased in in vivo studies compared to animal models such as mice and rats, thereby providing a reliable bridge for the future clinical applications of AgNPs.

Another limitation is the current lack of sufficient clinical data on the effects of AgNPs in humans. The majority of current clinical studies are based on limited sample sizes and fail to generate strong evidence on key issues such as long-term outcomes, optimal dosage intervals, and systemic toxicity. More extensive clinical data should be obtained to determine the long-term effects of AgNPs on human health, optimal dosage intervals, routes of administration, and treatment durations. Considering that systemic absorption of AgNPs may increase, especially in large-surface or open wounds, their potential toxicities should be carefully evaluated.

At this point, the green synthesis method stands out as a strong alternative both in terms of scientific studies and environmental sustainability, aiming to reduce the potential toxic effects of AgNPs and to enhance their biocompatibility. This synthesis method, which is less toxic compared to the chemical and physical synthesis methods of AgNPs, is a preferable option in clinical applications, especially in terms of human health and environmental safety. In the future, extensive studies should be conducted on the efficacy, stability, and long-term safety of AgNPs produced through the green synthesis method for wound healing.

On the other hand, the biological activity and safety of AgNPs are influenced by the synthesis method as well as by their physicochemical properties, including particle size, shape, and surface features. These parameters can significantly affect the level of interaction with cells, biological distribution, and toxicity. Similarly, treatment-specific variables such as the application dose, length of exposure, and route of administration of AgNPs are also among the key factors that influence their effectiveness. However, there are a limited number of studies in the current literature focusing on the standardization of all these parameters. Future research should focus on the systematic optimization of these parameters and on their development to be suitable for different wound types.

In conclusion, AgNPs are potential therapeutic agents for wound healing owing to their antibacterial, anti-inflammatory, antioxidant, and cell proliferation-promoting properties. However, to enable the safe and sustainable clinical application of this potential, it is essential to conduct a comprehensive evaluation of various factors, including toxicity, dosage, physicochemical properties, synthesis methods, and routes of administration. In the future, extensive studies that consider these parameters may enable the safer and more effective use of AgNPs in wound healing.

## Figures and Tables

**Table 1:** Selected In Vitro Studies on the Wound Healing Applications of AgNPs.

AgNP Type/Composite	Main Results	Physicochemical Properties	Reference
AgNPs-based topical cream (polysaccharide-capped AgNPs synthesized using the aqueous extract of *Punica granatum* polysaccharide)	-Increased keratinocyte proliferation and migration -Enhanced wound healing and closure -Antibacterial activity against *S. aureus*, *E. coli*, *K. pneumoniae*, *Salmonella typhi*, *Candida albicans* and *Candida krusei* -Low cytotoxicity on HaCaT cells	-Spherical shape -Size of 25 ± 5 nm (TEM)	[76]
AgNP + peptide (MT6 or CuTP1)	-Increased fibroblast migration -Long-term colloidal stability -Enhanced wound healing and closure rate in NIH/3T3 fibroblasts	-Spherical shape -Size of 126.90 ± 3.75 nm (MT6-AgNP), 181.60 ± 3.55 nm (CuTP1-AgNP) -SPR peak at 435 nm (MT6-AgNP), 453 nm (CuTP1-AgNP) -Zeta potential of 28.80 ± 0.46 mV (MT6-AgNP), −23.90 ± 1.21 mV (CuTP1-AgNP)	[77]
AgNPs-based topical ointment (Gelatin-coated AgNP + *T. procumbens* infused oil + probiotic biosurfactant)	-Antibacterial activity against *E. coli*, *S. aureus*, *P. aeruginosa* and *K. pneumoniae* -Enhancing wound healing and closure -Increased fibroblast migration -Inhibition of biofilm formation -No cytotoxicity on L929 fibroblasts -Minimal hemolytic activity on human RBCs	-Size of 4.3 ± 1.3 nm (TEM) -Mono-dispersed, polycrystalline structure	[78]
AgNPs + Alginate/gelatin hydrogel + plant extracts (Alg/gel + AgP)	-Increased fibroblast proliferation in V79 cells -Enhanced wound healing and closure -Strong antibacterial activity against *S. aureus* and *E. coli* -Low cytotoxicity for AgNP + plant extract composite (Alg/gel + AgP)	-Spherical shape -Hydrodynamic size of 244.1 nm -SPR peak at 416 nm -Zeta potential of −7.50 mV	[79]
AgNPs+ coated cotton-based dressing (green synthesized from the aqueous extract of *Scutellaria barbata*)	-Antibacterial activity against *S. aureus*, *E. coli*, *K. pneumoniae* and *P. aeruginosa* -Inhibition of biofilm formation -Increased fibroblast migration -Enhancing wound healing and closure -No cytotoxicity on L929 fibroblasts	-Spherical shape -Size of 20–40 nm -SPR peak at 400 nm	[80]
AgNPs (green synthesized from the leaf extract of *Rhizophora apiculata*)	-Promoted fibroblast migration -Accelerated wound healing and closure rate -Exhibited significant anti-inflammatory activity and antioxidant activity -Induced cytotoxicity in cancer cell lines -Showed low toxicity toward normal fibroblasts	-Irregular shape -Size of 35–100 nm (SEM), and 99 nm (DLS) -SPR peak at 459 nm -Zeta potential of −6 mV -Crystallinity confirmed by XRD	[81]
AgNPs + nanocollagen scaffold (*Catla catla* collagen + green-synthesized AgNPs from *Gymnema sylvestre*)	-Antibacterial activity against wound pathogens -Increased endothelial cell migration -Accelerated wound healing and closure rate on EA.hy 926 cell line -Dose-dependent cytotoxic effects	-Spherical shape -Size of 180–210 nm (TEM) and average size of 274 nm (DLS) -Collagen fiber diameter of ~500 nm (SEM) -SPR peak at 451 nm	[82]
AgNPs + Chitosan - powder form	-Antibacterial activity against *S. aureus* and *E. coli* -Enhanced wound healing and closure in L292 fibroblasts -Significant antioxidant activity -Dose-dependent cytotoxicity on HEK-293 cells	-Spherical shape -Size of 14–25 nm (TEM), 20–50 nm (SEM) and 55 ± 19.5 nm (DLS) -Zeta potential of +19.2 mV -SPR peak at 455 nm	[83]
AgNPs (green chemistry-based synthesis from *Azadirachta indica* leaf extract)	-Weak fibroblast migration in V79 cells -No significant enhancement in wound closure and rate -Antibacterial activity against MDR *E. faecalis* -Biofilm inhibition in planktonic and biofilm modes -No genotoxicity or DNA damage in normal fibroblasts -Moderate permeability in intestinal epithelial cell model -Induced apoptosis and cytotoxicity in HCT-116 colon cancer cells; no effect on MCF-7 cells	-Size range of 44.04 to 66.50 nm -Zeta potential of −55 mV	[84]
AgNPs embedded in gelatin/fucoidan nanogel	-Antibacterial activity against *S. aureus* and *E. coli* -Inhibition of biofilm formation -Enhanced fibroblast (L929) migration and proliferation -Accelerated wound healing and closure rate -No cytotoxicity on L929 fibroblasts	-Sphere-like structure with slight agglomeration -Size of 2–10 nm (TEM) and 10 nm (DLS) -Zeta potential of −28.45 ± 2.78 mV -SPR peak at 434 nm -Polydispersity index (PDI) of 0.155 ± 0.004	[85]

**Table 2:** Selected In Vivo and Clinical Studies on the Wound Healing Applications of AgNPs.

Study Type	AgNP Type/Composite	Main Results	Physicochemical Properties	Reference
In vivo—mice model	AgNPs-based topical ointment (green synthesized from *Trillium govanianum* rhizome extract)	-Enhancing wound healing and closure -Increased epithelialization -Significant anti-inflammatory activity -No dermal toxicity	-Not exactly spherical shape -Average size of ~27.94 nm (XRD) -SPR peaks at 295 nm and 350 nm	[106]
In vivo—mice model	AgNPs + vaseline-based topical ointment (green synthesized from *Cucumis sativus* pulp extract)	-Accelerated wound healing and closure rate -Significant anti-inflammatory activity and antioxidant activity -Increased fibroblast and fibrocyte concentration -Antibacterial activity against *S. aureus*	-Spherical and homogeneous shape -Size of 27–97 nm (SEM) -Crystallite size of ~4.39 nm (XRD) -SPR peak at 404 nm	[107]
In vivo—rat model	AgNPs + gelatin-based hydrogel dressing (green synthesized from *Mentha pulegium extract* under UV irradiation)	-Accelerated wound healing and closure rate -Significant antibacterial activity against *E. coli*, *S. aureus*, and MRSA -Reduced inflammatory cell infiltration -Promoted collagen deposition -Significant antioxidant activity -No cytotoxicity on HUVECs and no systemic toxicity in rat	-Spherical shape -Average size of 27.67 ± 0.05 nm (DLS) -SPR peak at 415 nm (at 410 nm at high pH) -Zeta potential of –19.0 mV	[108]
In vivo—rat model	AgNPs + vaseline-based topical nanoformulation (green synthesized from *Nepeta cataria* extract)	-Accelerated wound healing and closure rate -Promoting complete epithelialization -Increasing Type I collagen formation -Antibacterial activity against *E. coli*, *E. faecalis*, and *S. aureus* -Significant antioxidant activity	-Spherical shape -Size of 16.55–32.30 nm (TEM) -Size of ~15.74 nm (XRD) -SPR peak at 438 nm	[109]
In vivo—rat model	AgNPs + Carbopol-based gel (AgNPs–lactoferrin complexes using bovine lactoferrin)	-Accelerated wound healing and closure rate -Antibacterial activity against *E. coli* and *S. aureus* -Significant anti-inflammatory effect and antioxidant activity -No systemic toxicity -Observed hair regrowth, indicating skin biocompatibility	-Spherical shape -Size of 21.18 ± 3.6 nm (TEM) and 25.81 ± 2.31 nm (DLS) -Crystallite size of 16.4 nm (XRD) -SPR peak at 427 nm -Zeta potential of –21.7 mV	[110]
In vivo—rat model	AgNPs -based topical ointment formulation (green synthesized from *Juglans regia* L. pellicle extract)	-Accelerated wound healing and wound closure rate -Increased tensile strength -Reduced epithelialization time -Promoted collagen deposition and angiogenesis -Downregulated IL-1β levels -Upregulated TNF-α levels -No dermal irritation or inflammation	-Spherical shape -Average size of 108 nm (DLS) -PDI of 0.338 -SPR peak at 380–420 nm	[111]
In vivo—rat model	AgNPs (Gavage-administered)	-Accelerated wound healing and closure rate -Increased fibroblast concentration -Increased collagen deposition -Reduced inflammatory cells (PMNs, eosinophils, mast cells) -Moderate improvement in epithelialization and granulation tissue -Dose-dependent cytotoxic effects	-Spherical shape -Size of ~10 nm	[112]
In vivo—rat model	Curcumin-stabilized AgNPs + guar gum-based hydrogel	-Promoted fibroblast proliferation, migration, and collagen production in dermal cell culture -Low cytotoxicity in human dermal fibroblast cultures -Accelerated wound healing and closure rate -Reduced bacterial load at wound area -Enhanced fibroblast and capillary formation -Increased collagen deposition and re-epithelialization -Modulated expression of IL-6, EGF, FGF2, TGF-β1, Collagen-1, and Collagen-3	-Spherical shape -Size of 18.24 ± 4.20 nm (TEM) and 51.9 ± 0.8 nm (DLS) -PDI of 0.428 -Zeta potential of −25.2 ± 1.4 mV -SPR peak at 407 nm	[113]
In vivo—rat model	AgNPs aqueous suspen (green synthesized from marine *Bacillus subtilis* CBPPR1)	-Antibacterial activity against *S. aureus*, *E. coli*, and *K. pneumoniae* -Induced apoptosis and cytotoxicity in cancer cell lines -Accelerated wound healing and closure rate -Reduced pus formation and inflammation -No histopathological toxicity in liver and kidney tissues	-Spherical and oval shape -Size of 11.12–22.48 nm (TEM) and 265.4 nm (DLS) -SPR peak at 445 nm -Zeta potential of –22.5 mV	[114]
In vivo—rabbit model	AgNPs (synthesized from *Shilajit* extract)	-Accelerated bone healing and remodeling -Increased bone alkaline phosphatase (BAP) -Increased osteocalcin (OC) -Enhanced radiographic healing score	-Spherical shape -Average size of 250 ± 10.68 nm (TEM) and 372.3 ± 4.56 nm (DLS) -Zeta potential of −23.7 ± 1.22 mV -SPR peak at 480 nm	[115]
In vivo—rabbit model	AgNPs/chitosan (CS) + L-PRF scaffold	-Increased anastomotic bursting pressure -Reduced postoperative pain scores (BRPS) -Decreased adhesion formation -Lower stenosis degree -Enhanced collagen deposition and re-epithelialization -Promoted angiogenesis (increased VEGF and CD31 expression) -Improved histopathological healing scores -No systemic toxicity or adverse effects	-Spherical shape and polycrystalline structure (SAED) -Average size of 4.2 nm (TEM) -SPR peak at 420 nm -Zeta potential of +27.9 ± 9.35 mV	[116]
In vivo—rabbit model	AgNPs-based mouthwash	-Antibacterial activity against oral microflora -Enhancing wound healing and closure rate	-Spherical shape -Size of ~5.57 nm (DLS) -PDI of 0.3 -Zeta potential of low (stable suspension)	[117]
In vivo—rabbit and rat model	AgNPs + GVF/AGP-based hydrogel	*Rat model* -Accelerated wound healing and closure rate -Increased epidermal thickness -Decreased inflammatory cell infiltration -Increased expression of Col1a1, Col3a1, Egf, Fn1, Mmp1, Tgfb1, Vegf -Antibacterial activity against *S. aureus*, *E. coli*, *Burkholderia pseudomallei* *Rabbit model* -Minimal skin irritation -Transient mild erythema -No edema or persistent dermal damage	-Spherical or pseudo-spherical shape -Average size of 16.85 ± 5.81 nm -SPR peak at 441 nm	[118]
In vivo—mice model + Clinical study	AgNPs (green synthesis from gallic acid) + radiosterilized pig skin (RPS) + fibroblast & keratinocyte-based wound dressing	-Supporting fibroblast, keratinocyte, and mesenchymal stem cell (MSC) adhesion and viability -Inducing fibroblast growth factor (FGF) release from keratinocytes -Accelerated wound healing and closure rate -Increasing extracellular matrix (ECM) deposition -Antibacterial activity against Staphylococcus aureus biofilm -No systemic toxicity -Promoting complete re-epithelialization (clinical) -Improving elasticity, moisture, and pigmentation (clinical)	-Spherical shape -Size of ~10 nm (TEM, DLS) -Zeta potential of—42.3 mV -SPR peak at 420 nm	[119]
Clinic study	AgNPs-based topical cream (Kadermin)	-Accelerated wound healing and closure rate -Promoting re-epithelialization -Accelerated bacterial clearance from infected wounds -Broad-spectrum antibacterial activity against Gram-positive and Gram-negative wound bacteria -Reduced risk of new bacterial colonization -Reduced pain score -No local or systemic adverse effects	—	[120]
Clinic study	Nanocolloidal silver-based gel (SilvoGel)	-Significant improvement in ulcer area, exudate, and tissue type -Greater ulcer area reduction –Higher patient and physician satisfaction -Fewer local side effects (itching, irritation, discoloration)	—	[121]
Clinic study	AgNPs-based dressing (AgCoat ^®^)	-Reduced PUSH scores -Significant reduction in wound surface area -Reduced exudate levels -Increased granulation and epithelial tissue -Increased family satisfaction	—	[122]

## Data Availability

No new data were created or analyzed in this study.
